# Hypermethylation of PDX1, EN2, and MSX1 predicts the prognosis of colorectal cancer

**DOI:** 10.1038/s12276-022-00731-1

**Published:** 2022-02-15

**Authors:** Yeongun Lee, So Hee Dho, Jiyeon Lee, Ji-Hyun Hwang, Minjung Kim, Won-Young Choi, Jin-Young Lee, Jongwon Lee, Woochul Chang, Min Young Lee, Jungmin Choi, Tae-You Kim, Lark Kyun Kim

**Affiliations:** 1 Severance Biomedical Science Institute, Graduate School of Medical Science, Brain Korea 21 Project, Gangnam Severance Hospital, Yonsei University College of Medicine, Seoul, South Korea; 2 Interdisciplinary Program of Integrated OMICS for Biomedical Science, The Graduate School, Yonsei University, Seoul, South Korea; 3 Department of Biochemistry, College of Life Science and Biotechnology, Yonsei University, Seoul, South Korea; 4 Department of Biomedical Sciences, Korea University College of Medicine, Seoul, South Korea; 5 Department of Biology Education, College of Education, Pusan National University, Busan, South Korea; 6 College of Pharmacy, Research Institute of Pharmaceutical Sciences, Vessel-Organ Interaction Research Center (MRC), Kyungpook National University, Daegu, South Korea; 7 Department of Internal Medicine, Seoul National University Hospital, Department of Molecular Medicine and Biopharmaceutical Sciences, Graduate School of Convergence Science and Technology, Cancer Research Institute, Seoul National University, Seoul, South Korea

**Keywords:** Prognostic markers, Methylation analysis

## Abstract

Despite numerous observations regarding the relationship between DNA methylation changes and cancer progression, only a few genes have been verified as diagnostic biomarkers of colorectal cancer (CRC). To more practically detect methylation changes, we performed targeted bisulfite sequencing. Through co-analysis of RNA-seq, we identified cohort-specific DNA methylation markers: CpG islands of the intragenic regions of PDX1, EN2, and MSX1. We validated that these genes have oncogenic features in CRC and that their expression levels are increased in correlation with the hypermethylation of intragenic regions. The reliable depth of the targeted bisulfite sequencing data enabled us to design highly optimized quantitative methylation-specific PCR primer sets that can successfully detect subtle changes in the methylation levels of candidate regions. Furthermore, these methylation levels can divide CRC patients into two groups denoting good and poor prognoses. In this study, we present a streamlined workflow for screening clinically significant differentially methylated regions. Our discovery of methylation markers in the PDX1, EN2, and MSX1 genes suggests their promising performance as prognostic markers and their clinical application in CRC patients.

## Cancer: Mapping malignant modifications

An experimental strategy for detecting patterns of DNA modification reveals gene-specific alterations associated with worse outcomes in colorectal cancer patients. Many genomic regions undergo a process of chemical modification called methylation, which can strongly affect the expression of nearby genes. Many cancers exhibit abnormal methylation, and South Korean researchers led by Tae-You Kim of Seoul National University and Lark Kyun Kim of Yonsei University College of Medicine, Seoul, have developed a strategy for identifying such tumor-specific modifications. They identified a trio of genes that undergo excessive methylation in colorectal cancer, and show that this ‘signature’ is associated with more advanced metastatic tumors and shorter overall survival. The results from this study could give clinicians an additional diagnostic tool, and highlight the potential utility of performing such methylation profiling in other cancer types.

## Introduction

Colorectal cancer (CRC) is the third most common cancer worldwide, accounting for the second-highest mortality in 2020^1^. CRC is widely known to occur due to the accumulation of genetic and epigenetic alterations. Several molecular pathways involved in the onset and development of CRC have been identified, including the adenoma–carcinoma pathway (also called the chromosomal instability sequence), the serrated neoplasia pathway, and microsatellite instability (MSI)^2,3^. The adenoma–carcinoma pathway accounts for 70–90% of CRC cases and is generally initiated by *APC* mutations, followed by *KRAS* activation or loss of *TP53* function. Conversely, the serrated neoplasia pathway develops via *KRAS* and *BRAF* mutations, and epigenetic dysregulation is uniquely distinguished by the CpG island methylator phenotype (CIMP). MSI typically occurs with Lynch syndrome, mainly due to mismatch repair (MMR) gene inactivation^4–7^.

In the United States, 20% of the patients diagnosed with CRC in 2020 had metastatic CRC (mCRC)^8^. Early detection of CRC is highly critical because adjuvant chemotherapy is no longer efficient and survival rates are significantly decreased for patients with CRC diagnosed at late cancer stages (stage III or IV)^9,10^. With the clinical need for early CRC diagnosis, many diagnostic and prognostic markers based on genomic alterations have been comprehensively studied. Unfortunately, few markers are used in marker development to predict the probability of metastasis or recurrence despite their unmet clinical needs.

Among the epigenetic modifications in mammals, DNA methylation plays a key role in regulating gene expression. This epigenetic regulation affects tumor suppressor gene and oncogene expression, which may lead to cancer progression. This mode of action is slightly different among cancer types, and DNA methylation markers have been extensively established in CRC. Because of the hypomethylation and activation of repetitive sequences, such as long interspersed nuclear element-1 and Alu repeats, genomic instability is thought to occur and could boost CRC initiation^11–13^. Conversely, researchers also found a panel of genomic regions and genes aberrantly hypermethylated at the promoter regions in some CRCs, which was later identified as a type of CRC called CIMP^14^. In general, gene expression is decreased when DNA hypermethylation occurs in the promoter of a gene; thus, hypermethylated genes of the CIMP are thought to function as tumor suppressors.

Despite numerous observations regarding the relationship between DNA methylation changes and cancer progression, only a few genes, such as *SEPT9* (Epi proColon), *NDRG4*, and *BMP3* (Cologuard), have been verified as diagnostic CRC biomarkers and have been approved for commercialization via diagnostic kits^15–17^. While the surprising lack of translation into commercially viable DNA methylation-based biomarkers can be explained by methodological and experimental hurdles^18^, the cornerstone of developing DNA methylation-based biomarkers is the selection of ideal genomic locations, that is, CpG islands (CGIs) and specific CpG sites^19^. For example, in several investigations, DNA methylation in the promoter region of *GSTP1* has been identified as a promising diagnostic marker for hepatocellular carcinoma but with conflicting variation in terms of its specificity. It was later discovered that this variability resulted from differences in the CpG sites of the 5′ region of the *GSTP1* promoter used for measuring DNA methylation levels^20^. In other words, this suggests that detection sensitivity and clinical relevance may vary depending on how the CpG sites within the same CpG island are selected.

To discover clinical biomarkers based on next-generation sequencing technology, Illumina Infinium 450 K or 850 K array-based detection methods have been used for massive data generation by The Cancer Genome Atlas (TCGA)^21^. This method enables us to screen and observe the methylation levels of various genes in cancer cells. Whole-genome bisulfite sequencing has emerged as a powerful method that determines DNA methylation levels on a genome-wide scale but is limited by its high cost and the time required to obtain a statistically sufficient sample size. Targeted sequencing technology has emerged as a tool for the high-throughput sequencing of genomic regions of interest. To increase the specificity of the quantification of DNA methylation, targeted sequencing has been applied to bisulfite sequencing. In detail, targeted bisulfite sequencing utilizes probes designed to bind and capture target regions for PCR-based enrichment. These capturing and enrichment steps allow us to obtain a reliable depth of DNA methylation data at the CpG site level. This method has the advantage of selecting the largest difference in DNA methylation levels and the most clinically relevant CpG sites among CpG islands or other genomic regions^22^. However, a more straightforward methylation method, methylation-specific polymerase chain reaction (MS-PCR, MSP), has been developed and used to validate the methylation status^23^. This method offers a time- and cost-effective way of observing methylation in target regions, while designing primers and optimizing PCR conditions are relatively laborious^24,25^.

This study presents our streamlined workflow for screening clinically significant differentially methylated regions and proposes primer sequences for qMSP employed as a time- and cost-effective DNA methylation detection method for clinical applications. We preliminarily selected tumor-specific methylated regions from the Infinium 450 K microarray data downloaded from TCGA. We then generated hybrid capture-based targeted bisulfite sequencing data from a South Korean CRC patient cohort at Seoul National University Hospital (SNUH). We identified cohort-specific DNA methylation markers in the CpG islands of *PDX1, EN2*, and *MSX1* and validated tumor-specific hypermethylation levels of these three genes via optimized qMSP methods with highly sensitive primer sets. We also assessed their prognostic prediction performance and found that subgroups based on the methylation status of the identified biomarkers displayed significantly different recurrence and survival rates in CRC patients. Our discovery of methylation markers in the *PDX1, EN2*, and *MSX1* genes suggests their potential as prognostic markers and their clinical application in CRC patients.

## Materials and methods

### Analysis of Infinium HumanMethylation450 BeadChip data from TCGA

To select candidate genomic DNA regions for targeted bisulfite sequencing, Infinium HumanMethylation450 BeadChip data from TCGA were downloaded from the repository of five major gastrointestinal cancers, namely, colon adenocarcinoma (COAD), rectal adenocarcinoma (READ), liver hepatocellular carcinoma (LIHC), stomach adenocarcinoma (STAD), and pancreatic adenocarcinoma (PAAD), via the Genomic Data Commons (GDC) Data Portal (https://portal.gdc.cancer.gov/). The beta value of each CpG site was averaged to represent the methylation value of their matched CpG island in accordance with the human genome ref. ^19^ (hg19). The CpG island methylation values of healthy tissue samples were then averaged, and methylation differences between the tumor samples and the average of the healthy tissue samples were tabulated. Finally, we shortlisted CpG islands that displayed methylation differences between normal and tumor tissues greater than or equal to 20% in more than 20% of the total patients. According to these criteria, the total number of target regions was 18,834 (10,754 CpG islands), and the total length of the regions was 23,533,457 bp.

### Design of the hybridizing probe pool

The probe pool was designed according to the manufacturer’s instructions. Basic information regarding our target genome is as follows: Application—SeqCap Epi, Organism—*Homo Sapiens*, Genomic builds—hg19/GRCh37. This was followed by data input in an appropriate bed format into NimbleDesign Software (version 4.3; Roche Diagnostics, Rotkreuz, Switzerland).

### Colorectal tumor and adjacent healthy specimens

A total of 104 colorectal tumors and their adjacent healthy tissues were obtained from Seoul National University Hospital (SNUH; Seoul, Korea). The use of samples was approved by the Institutional Review Board of Seoul National University Hospital and carried out in accordance with the ethical standards and guidelines of the institution (IRB number: 1608-040-784).

### Sample preparation for targeted bisulfite sequencing

Genomic DNA (1 µg) was used to prepare a single targeted bisulfite sequencing library. All genomic DNA of healthy and tumor samples were sheared using a focused ultrasonicator (M220; Covaris, Massachusetts, USA). The quality, quantity, and fragment size (major peak in 250–300 bp) of sheared genomic DNA were verified using a 2100 Bioanalyzer system (G2939BA; Agilent Technologies, California, USA) prior to library preparation. Sheared genomic DNA was then processed through end repair, A-tailing (Kapa Library Prep Kit for Illumina NGS Platform, 7137974001; Roche Diagnostics), and sequencing adaptor ligation steps (SeqCap Adapter Kit A, 7141530001; Roche Diagnostics). After clean-up with Agencourt AMPure XP beads (A63880, Beckman Coulter, California, USA), the DNA library was bisulfite-converted using the EZ DNA Methylation-Lightning Kit (D5031; Zymo Research, California, USA) and amplified via precapture polymerase chain reaction (PCR) using KAPA HiFi HotStart Uracil+ ReadyMix (NG SeqCap Epi Accessory Kit, 7145519001; Roche Diagnostics) with Pre-LM-PCR Oligo. The quality of the amplified, bisulfite-converted library samples and their sizes (main peak in 250–300 bp) were verified using a Bio-Analyzer. One microgram of each amplified, bisulfite-converted library was then combined in sets of SeqCap Epi universal and indexing oligos and bisulfite capture enhancer (SeqCap EZ HE-Oligo Kit A, 6777287001; Roche Diagnostics). Each pool was subsequently lyophilized using a DNA vacuum concentrator (Modulspin 31; Hanil Science Co, Ltd., Daejeon, South Korea). The dried components were resuspended in hybridization buffer (SeqCap Epi Hybridization and Wash Kit, 5634253001; Roche Diagnostics) and hybridized with the probe pool (SeqCap Epi Choice S, 7138938001; Roche Diagnostics) for 72 h at 47 °C in a thermocycler with a heated lid at 57 °C. Following incubation, libraries were captured (SeqCap Pure Capture Bead Kit, 6977952001; Roche Diagnostics) in a 47 °C water bath and purified at room temperature. Captured bisulfite-converted libraries were amplified via postcapture PCR and then washed with AMPure XP beads. The quality and size (single peak in 250–300 bp) of the libraries were checked using a bioanalyzer, and samples that passed quality control were sequenced on a HiSeq 2500 instrument (Illumina, California, USA) in paired-end mode.

### Preprocessing and preliminary screening of targeted bisulfite sequencing data

Trim Galore (version 0.5.0) was used to remove the adaptor sequences from the targeted bisulfite sequencing data based on the human CpG island reference hg19 file. Bismark was used to align sequencing reads with Bowtie2. The sort and index commands from SAMtools were used. The number of methylated and unmethylated cytosines at each CpG site was listed using a Bismark methylation extractor from post-indexed data, and only those 10× or higher were selected for downstream analysis. Finally, the methylation values of CpG sites included in the same CpG island were calculated by averaging the methylation value based on the hg19 reference file. The following analyses were performed based on the assumption that the averaged value represents each respective CpG island. Targeted bisulfite sequencing data were screened for targets in which DNA methylation increased or decreased by >30% in tumor samples compared with healthy tissue samples in >50% of the 90 patients. In addition, hypermethylated CpG islands in tumor samples were further filtered to retrieve regions that showed <30% DNA methylation in the healthy tissue samples and 50% or greater DNA methylation in the tumor samples. Conversely, hypomethylated CpG islands, in which the average DNA methylation was <30% in tumor samples and greater than 50% in the healthy tissue samples, were selected. Finally, we selected CpG islands where the mean DNA methylation in healthy tissue samples and tumor samples differed by >30%.

### Analysis of targeted bisulfite sequencing data

To analyze the CpG site methylation levels in candidate CpG islands from healthy tissue and tumor samples, beta values of CpG sites in candidate CpG islands were extracted using the tabix program of SAMtools (version 1.9), and only the beta values of cytosines in the same strand of adjacent genes were used in the subsequent analysis to identify the optimal MSP target sites. To filter out the low-quality sequencing data, only sequencing data in which the methylation levels of CpG sites were present in more than 1/3 of the total CpG sites in each CpG island were used. Hierarchical clustering with Canberra distance was applied to the methylation level of each sample using the pheatmap package (version 1.0.12) in R software. Line graphs were also drawn with the same methylation data using ggplot2 (version 3.3.3) and ggsci (version 2.9) in R software. To display the methylation differences of candidate CpG islands between healthy tissue and tumor samples, hierarchical clustering with Manhattan distance was conducted using pheatmap. Clustering of CRC patients was performed with the methylation data of the three candidate CpG islands in *PDX1*, *EN2*, and *MSX1*. Using IGV, the data regarding the average methylation levels of genes in healthy and tumor tissues were visualized in tandem with the CpG island and CpG site information.

## Results

### Identification of differentially methylated regions in CRC tissues by targeted bisulfite sequencing

**Fig. 1:**
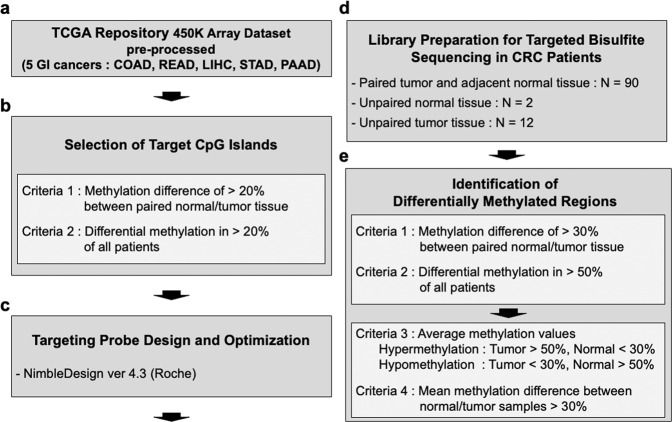
Overall workflow for cohort-specific DNA methylation biomarker selection in colorectal cancer. **a** Illumina Infinium 450 K array data of five major gastroenterological cancers (COAD, READ, LIHC, STAD, and PAAD) downloaded from TCGA were preprocessed. **b** Then, 10,754 differentially methylated CpG islands (CGIs) were shortlisted from processed 450 K array data based on our criteria. **c** The hybridizing probe pool targeting selected CGIs was designed using NimbleDesign. **d** Targeted bisulfite sequencing was conducted for 104 CRC patients from the South Korean cohort, of which 90 samples were paired tumor-adjacent healthy tissue sets, while two healthy samples and ten tumor samples were unpaired. **e** Generated targeted bisulfite sequencing data were analyzed to select differentially methylated regions (DMRs) in tumors relative to healthy tissues, giving rise to 40 DMRs for further examination.

To observe methylation levels in CRC and other types of cancers, we collected 450 K microarray data of five cancer types (COAD, READ, LIHC, AD, and PAAD) from TCGA (Fig. 1a). The beta value of each CpG site was averaged to represent the methylation value of their matched CpG island in accordance with the human genome ref. ^19^ (hg19). The selected CpG islands were further filtered using two criteria. One was that the difference in methylation values between healthy and tumor tissues should be more than 20%, and the other was that such a difference should be present in >20% of cancer patients. Therefore, we obtained 10,754 differentially methylated CpG islands (Fig. 1b and Supplementary Fig. 1). The selected CpG islands were designed to probe the pool using NimbleDesign (Roche), a software that predicts the coverage of the input sequence and optimizes the probe design according to its criteria so that the probe pool captures the target regions more efficiently (Fig. 1c).

Next, we performed bisulfite sequencing using the probe pool in CRC tissues. To do this, we obtained genomic DNA from the tissues of 104 Korean CRC patients (90 paired tumors and adjacent healthy tissues, an additional two healthy tissues, and 12 tumor tissues). Targeted bisulfite sequencing libraries were prepared according to the manufacturer’s instructions (Roche) (Fig. 1d and Supplementary Fig. 2), and sequencing was performed. Through targeted bisulfite sequencing of the 194 CRC tissues, we obtained the beta values of each CpG site, which were averaged to constitute the methylation value of their matched CpG island (Supplementary Fig. 3). After obtaining the methylation values of CpG islands, we applied more stringent criteria to our data. First, the difference in the methylation values of CpG islands between paired healthy and tumor tissues (i.e., from the same patient) had to be >30%. Second, this difference had to be present in >50% of the patients. Third, even if the difference in methylation values between healthy and tumor tissues was >30%, the lower value had to be <30%, enabling the easy optimization of MSP by maximizing the signal-to-noise ratio. Finally, to identify the differentially methylated regions that are not specific to some patients, after calculating the overall average of healthy and tumor tissues, the regions with a difference of more than 30% were selected (Fig. 1e).

Thus, we ultimately identified 40 differentially methylated CpG islands consisting of 35 hypermethylated regions and 5 hypomethylated regions in tumor tissues. For instance, the genomic location of chromosome 7:27,147,589–27,148,389 is the intragenic region of HOXA3, where 67 CpG sites are located. On average, the methylation level in this region was 29% in healthy tissues and 78.7% in tumor tissues. This difference was observed in 83.3% of CRC patients (75 out of 90) (Table 1).

**Table 1:** Candidate CpG islands and their matched genes selected from the targeted bisulfite sequencing data of 90 CRC patients are listed, and information pertaining to the genomic and functional location of CpG islands and their adjacent gene name is provided.

CGI_location	CGI_info	Gene	30%_Diff	McoM	McaM	(McaM-McoM)
chr7:27147589–27148389	intragenic	HOXA3	83.3% (75/90)	29.0	78.7	49.7
chr7:27146069–27146600	intragenic	HOXA3	82.2% (74/90)	26.0	74.0	48.0
chr19:49669275–49669552	intragenic	TRPM4	81.1% (73/90)	24.2	73.7	49.5
chr2:54086776–54087266	promoter	GPR75-ASB3	80% (72/90)	23.9	74.3	50.3
chr1:200010625–200010832	intragenic	NR5A2	78.9% (71/90)	9.1	57.7	48.7
chr13:28498226–28499046	intragenic	PDX1	72.2% (65/90)	9.1	55.0	45.9
chr5:140857864–140858065	intragenic	PCDHGA2	72.2% (65/90)	17.3	62.8	45.5
chr7:27182613–27185562	promoter	HOXA-AS3	71.1% (64/90)	21.4	62.6	41.2
chr19:48918115–48918340	intragenic	GRIN2D	69.9% (58/83)	10.7	53.1	46.2
chr5:140864527–140864748	promoter	PCDHGA2	68.9% (62/90)	9.1	52.3	43.1
chr5:134363092–134365146	intragenic	PITX1	67.8% (61/90)	21.5	59.8	38.3
chr7:158936507–158938492	promoter	VIPR2	65.6% (59/90)	12.4	50.1	37.7
chr6:62995855–62996228	promoter	KHDRBS2	63.3% (57/90)	11.7	51.3	39.6
chr6:10398573–10398812	intragenic	TFAP2A	63.3% (57/90)	16.1	53.0	36.9
chr7:27143181–27143479	intergenic	—	63.3% (57/90)	26.0	62.6	36.7
chr7:24323558–24325080	promoter	NPY	63.3% (57/90)	16.5	52.7	36.2
chr8:97171805–97172022	promoter	GDF6	63.3% (57/90)	19.8	53.5	33.7
chr13:53313127–53314045	promoter	CNMD	62.2% (56/90)	15.6	50.9	35.3
chrX:142721410–142722958	promoter	SLITRK4	60.7% (54/89)	19.2	54.8	35.5
chr7:155255098–155255311	intragenic	EN2	60% (54/90)	17.0	52.2	35.2
chr13:102568425–102569495	promoter	FGF14	60% (54/90)	15.6	50.6	35.0
chrX:66766037–66766279	intragenic	AR	58.9% (53/90)	20.3	55.8	35.5
chr9:37002489–37002957	promoter	PAX5	58.9% (53/90)	22.1	56.3	34.1
chrX:101906001–101907017	promoter	ARMCX5-GPRASP2	57.8% (52/90)	21.6	58.2	36.6
chr4:111549879–111550203	intragenic	PITX2	57.8% (52/90)	22.9	53.7	30.8
chr4:4864456–4864834	intragenic	MSX1	57.3% (51/89)	29.7	64.3	35.3
chr8:72753874–72754755	promoter	MSC	56.7% (51/90)	26.7	58.7	32.0
chr19:46915311–46915802	intragenic	CCDC8	55.6% (50/90)	17.7	52.1	34.5
chr8:130995921–130996149	intragenic	FAM49B	54.4% (49/90)	20.9	53.1	32.1
chr2:98962873–98964187	promoter	CNGA3	54.4% (49/90)	19.6	51.7	32.1
chr2:5836068–5837643	intragenic	SOX11	54.4% (49/90)	20.8	51.7	30.9
chr11:65359292–65360328	intragenic	EHBP1L1	53.3% (48/90)	26.6	58.0	31.4
chr6:108495654–108495986	intragenic	NR2E1	53.3% (48/90)	21.5	52.0	30.5
chr1:120905971–120906396	promoter	HIST2H2BA (H2BP1)	53.3% (48/90)	28.8	59.1	30.3
chr13:70681732–70682219	promoter	KLHL1	50% (45/90)	25.1	55.5	30.4
**CGI_location**	**CGI_info**	**Gene**	**30%_Diff**	**McoM**	**McaM**	**(McaM-McoM)**
chr16:87441387–87441671	intragenic	ZCCHC14	78.9% (71/90)	77.98	28.81	−49.17
chr7:5342299–5342599	intragenic	SLC29A4	77.8% (70/90)	73.15	26.40	−46.75
chr20:33762403–33762774	intragenic	PROCR	66.7% (60/90)	68.94	29.90	−39.04
chr1:235805318–235805771	intragenic	GNG4	56.7% (51/90)	62.69	29.03	−33.66
chr2:233925091–233925318	promoter	INPP5D	57.8% (52/90)	52.94	20.31	−32.63

The proportion of patients whose differences in CpG island methylation levels are significantly different between healthy tissues and cancer tissues was calculated along with the average methylation levels in healthy tissue or tumors and their difference in values. According to our criteria, we found 35 hypermethylated CpG islands and five hypomethylated CpG islands in tumors. *McoM* the mean of control (healthy) methylation, *McaM* the mean of case (cancer) methylation.

### Selection of candidate genes for developing CRC biomarkers

The methylation location plays an important role in the correlation between methylation states and gene expression^19,26–28^. However, while it is well accepted that hypermethylation in the promoter region inhibits gene expression^29^, the effect of methylation of the intragenic regions on gene expression is still controversial^30–36^.

**Fig. 2:**
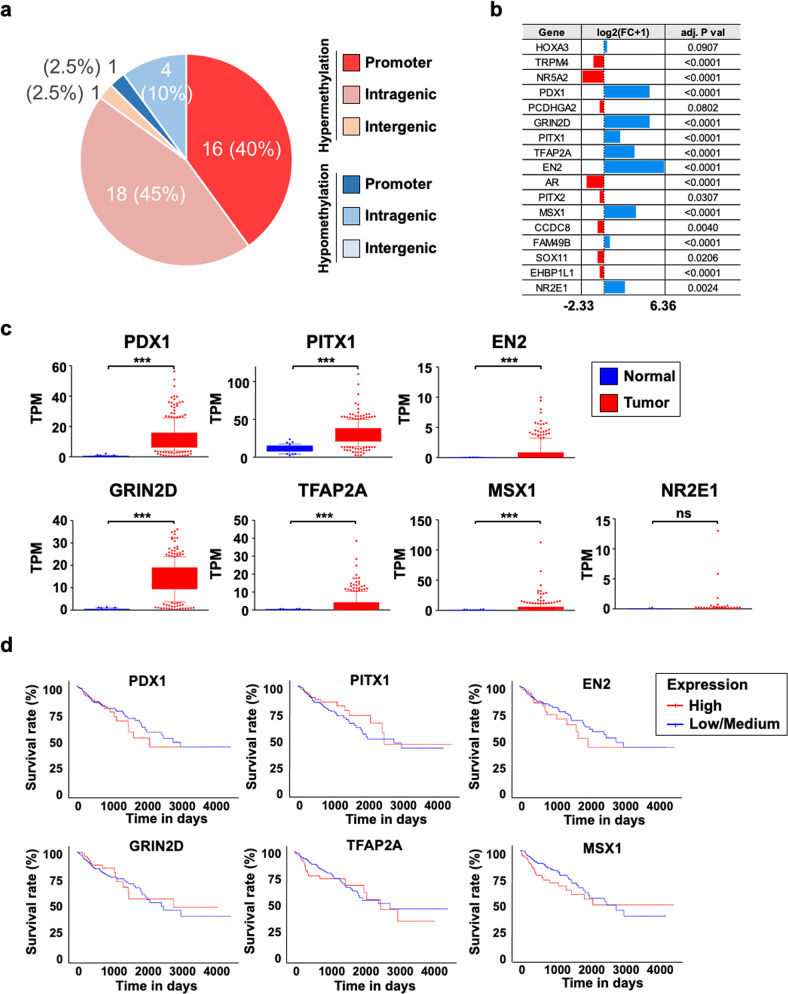
Streamlining of candidate DNA methylation biomarker genes based on differential gene expression and correlation with CRC patient survival outcomes. **a** Genomic location analysis of differentially methylated CGIs in targeted bisulfite sequencing data indicates that most hypermethylated regions are evenly distributed between the promoter and intragenic regions, while a larger proportion of hypomethylated regions are in intragenic regions. Our focus was on hypermethylated intragenic regions. **b** The expression data (read counts) downloaded from TCGA were examined to identify upregulated genes in tumor samples relative to healthy tissue samples. Downloaded RNA-seq data were processed with DESeq2 in R. **c** Gene expression representation of seven upregulated candidate genes in terms of TPM. Their differential expression status was further verified, and genes with nonsignificant differences were omitted from downstream analysis. Expression data between normal and tumor tissues were downloaded from TCGA, and TPM values were derived by multiplying the scaled-estimate value of RNA-seq data by 10^6^. Significance levels are presented as ns: nonsignificant, *p < 0.05, **p < 0.01, ***p < 0.001. **d** Kaplan–Meier survival plots (generated by the UALCAN database) of the six upregulated genes indicated the difference between patients with high expression of the shortlisted genes (top 25%) and patients with low or medium expression (bottom 75%). Gene expression and clinical data were based on TCGA-COAD.

When we looked at the locations of our 40 differentially methylated CpG islands in terms of the promoter, intragenic, and intergenic regions, we observed that among the 35 hypermethylated regions in the tumor, 16 CpG islands were in the promoter region, 18 were in the intragenic region, and 1 was in the intergenic region. Among the five hypomethylated regions, one was in the promoter region, and four were in the intragenic region (Fig. 2a and Table 1).

After identifying the 40 differentially methylated CpG islands in CRC tissues, we next wanted to develop a system to detect methylation states in these regions in association with cancer status. To do this, we examined the regions whose methylation changes have a direct correlation with the expression changes of the related genes. We speculated that it would be much easier to detect the changes if both methylation and gene expression are increased in tumor tissues compared with healthy tissues because it is easy to determine what exists from what does not, but it is not easy to quantify its importance. Therefore, we were interested in the hypermethylated regions, particularly in intragenic regions, because it is difficult to connect the intergenic region to gene expression, and hypermethylation in the promoter is well accepted to be related to decreased gene expression. To examine gene expression, we took advantage of the TCGA RNA-seq dataset of colon adenocarcinoma (Supplementary Fig. 4). Among the 18 hypermethylated intragenic regions, two regions were contained in the HOXA3 gene, so we sought to check the expression of 17 genes. According to the count data analyzed by DESeq2, the expression of only seven genes (*PDX1, GRIN2D, PITX1, TFAP2A, EN2, MSX1*, and *NR2E1*) was increased by more than two times in tumors (Fig. 2b). To ascertain the level of upregulation of these seven genes, we also checked the expression of other candidate genes along with that of the seven genes in terms of the TPM value and then excluded NR2E1 due to lack of statistical significance (Fig. 2c and Supplementary Fig. 5). To further confirm the relationship between methylation changes and gene expression using Pearson and Spearman correlations, we used the Infinium HumanMethylation 450 BeadChip data and RNA sequencing data obtained from the same samples from TCGA-COAD. We found that the methylation level of the promoter CpG islands was inversely correlated with the expression of matched genes in tumor samples, regardless of whether it was significant (Supplementary Fig. 6). In contrast, the methylation of some intragenic CpG islands had a positive correlation with matched gene expression (Supplementary Fig. 7). That is, *PDX1*, *EN2*, and *MSX1* had higher expression levels in tumors than in normal tissues, and methylation and expression levels were positively correlated (Fig. 2b, c and Supplementary Figs. 5–7).

Next, we examined the relationship between the expression of the six genes obtained and the survival rate of CRC patients. The greater the role of abnormally expressed genes in tumor tissues, the lower the survival rate is. According to UALCAN analysis^37^, high expression of *PDX1, EN2*, and *MSX1* was negatively correlated with patient survival (Fig. 2d). Therefore, we decided to focus on examining these three genes.

### Overexpression of PDX1, EN2, or MSX1 promotes cell proliferation and invasion in human colon cancer cells

Pancreatic and duodenal homeobox 1 (PDX1) is a critical transcription factor for pancreatic development and beta-cell maturation^38^. PDX1 is overexpressed in pancreatic cancer cells, but its role is different at each cancer stage^39–41^. Although PDX1 has already been reported as a potential cancer marker in CRC, it is based on the observation of PDX1 expression in cancer cells, and its role has not been studied in detail. Homeobox protein engrailed-2 (EN2) is a homeobox-containing transcription factor regulating many developmental stages^42^. Very recently, EN2 was reported to play an oncogenic role in tumor progression via CCL20 in CRC^43^. Msh homeobox 1 (MSX1) is also a homeobox-containing transcription factor. MSX1 has been suggested as an mRNA biomarker for CRC, but this suggestion was based on observations, and to our knowledge, its role has never been demonstrated at the cellular level in CRC^44^.

**Fig. 3:**
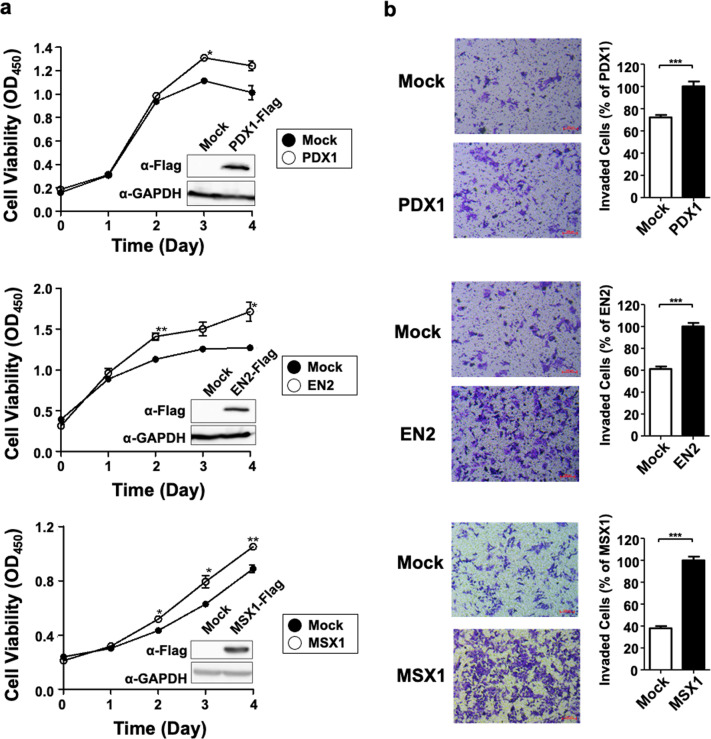
Selected candidate DNA methylation biomarker genes drive oncogenic properties by promoting cell proliferation and migration in vitro. **a** The cell proliferation test with CCK-8 reagent indicated that overexpression of PDX1, EN2, and MSX1 promotes proliferation of the HCT116 colon cancer cell line. The overexpression of each gene was verified through FLAG-tag capture. **b** Transwell invasion assays with HCT116 cells overexpressing PDX1, EN2, and MSX1 were conducted, and invading cells were stained with crystal violet. Overexpression of *PDX1*, *EN2*, and *MSX1* was found to accelerate migration and confer invasive properties.

As previously mentioned, we wanted to develop a system that identifies the methylation changes of related genes that play a role in CRC. Although a literature search suggested a role for each gene in CRC, we wanted to be more confident. Thus, we transiently transfected each gene into the HCT116 colon cancer cell line and then checked the status of the cells. Proliferation was determined using CCK-8, a colorimetric reagent that indicates cell viability. Overexpression of *PDX1*, *EN2*, and *MSX1* increased cell proliferation (Fig. 3a). In addition, when we performed the Transwell assay, we observed that *PDX1*, *EN2*, and *MSX1* significantly promoted HCT116 cell migration (Fig. 3b).

Overall, we concluded that since the overexpression of *PDX1*, *EN2*, and *MSX1* is directly related to the proliferation and migration of CRC cells, if the methylation changes in the intragenic regions of these genes are correlated with changes in gene expression, the detection of methylation changes in our marker regions would be able to predict cellular conditions.

### Design of MSP primers for the optimal detection of methylation changes

To detect the methylation changes in our marker regions, we decided to set up a qMSP for each region, but factors had to be considered first. Since MSP is a PCR-based experiment, the choice of primer region is very important. If each of the forward and reverse primers has as many CpG sites as possible, the ideal methylation difference between healthy and tumor tissue is large. However, because it would be preferred to perform PCR of methylated primers with unmethylated primers in the same machine, too many CpG sites may cause a Tm difference between methylated and unmethylated primers. Last, we attempted to make the amplicon length 100–160 bp because longer products may not be efficiently amplified. Overall, after many trials and errors, we decided that the forward and reverse primers had at least six CpG sites in total, the Tm of each primer was 55–60 °C, and the amplicon length was 100–160 bp.

**Fig. 4:**
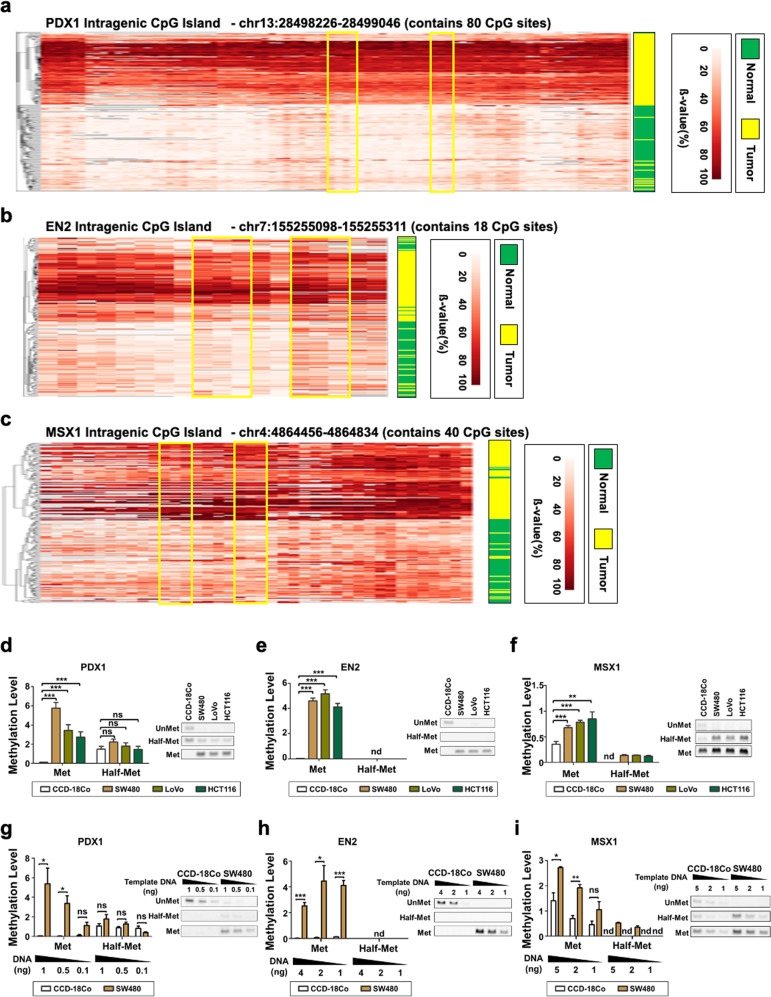
Optimized benchmark for primer-binding site selection and primer design in methylation-specific PCR (MSP). **a**–**c** MSP-targeting genomic regions in the intragenic CpG islands of *PDX1* **a**, *EN2* **b**, and *MSX1* **c** are boxed in yellow. Hierarchical clustering of healthy tissue and tumor samples of targeted bisulfite sequencing data confirmed the hypermethylation of each target region in the tumor relative to healthy tissues. Each column corresponds to the *cytosine* of CpG sites within the respective intragenic CpG islands of *PDX1*, *EN2*, and *MSX1*. Low-quality sequencing data were then filtered out**. d**–**f** The efficacy of methylation detection and quantification of manually designed MSP primers were validated in vitro, in which three colon cancer cell lines (SW480, LoVo, HCT116) and one healthy colon cell line (CCD-18Co) were used. Agarose gel electrophoresis of quantitative MSP (qMSP) products also confirmed the methylation level detection efficacy of the designed primers for *PDX1*, *EN2*, and *MSX1***. g**–**i** qMSP with varying CCD-18Co and SW480 template DNA quantities was conducted to verify DNA quantity-dependent signal changes of **g** *PDX1*, **h** *EN2*, and **i** *MSX1* methylation. Met: MSP primer that binds to genomic DNA where all the target CpG sites are methylated. Half-Met: the MSP primer that binds with genomic DNA where some of the target CpG sites are methylated. Unmet: MSP primer that binds with genomic DNA where all the target CpG sites are not methylated. nd: not determined. *p < 0.05, **p < 0.01, ***p < 0.001.

To design MSP primers specifically for the intragenic CpG island of *PDX1* (chr13:28,498,226-28,499,046), we examined the methylation changes of 80 individual CpG sites in that region. Although most CpG sites had large differences in methylation changes between tumor and healthy tissues, in an effort to identify the region that satisfies our criteria, we designed MSP primers according to the heatmap and the line graph of the methylation level for each CpG site in the candidate CpG islands (Fig. 4a and Supplementary Fig. 8a). Since we were interested in the methylation level of the same strand of the target CpG island, we mainly focused on the methylation level of CpG sites on the sense strand. The forward primer for *PDX1* has four CpG sites, and the reverse primer has three CpG sites. The beta value of these seven CpG sites was approximately 10% in normal tissues but 70% in tumor tissues on average. The amplicon size was 126 bp and 123 bp, and the Tm was 55–57 °C (Fig. 4a and Supplementary Fig. 8a). For EN2 and MSX1, MSP primers were designed through similar efforts. In brief, the forward primer and the reverse primer for EN2 had three CpG sites. The beta value of the six CpG sites was approximately 10% in healthy tissues but 70% in tumor tissues on average. The amplicon sizes were 127 bp and 112 bp, and the Tm was 57–58 °C (Fig. 4b and Supplementary Fig. 8b). The forward primer and the reverse primer for MSX1 had three CpG sites. The beta value of the six CpG sites was approximately 10% in healthy tissues but 70% in tumor tissues on average. The amplicon sizes were 151 bp and 144 bp, and the Tm was 55–57 °C (Fig. 4c and Supplementary Fig. 8c).

### MSP primers efficiently detect the methylation states of the region of interest

Next, we wanted to confirm whether our MSP primers properly detected methylation levels. Since our MSP primers had a total of six or seven CpG sites, we not only made a primer set that retained cytosine (methylation primers) or changed all cytosine to thymine (unmethylated primers) but also created a primer set that changed only half of the cytosine to thymine (half-methylation primers). Using these primers, we performed qPCR with bisulfite-treated genomic DNA from the CCD-18Co normal colon cell line and the SW480, LoVo, and HCT116 colon cancer cell lines.

In each CpG island, the methylation primer gave a PCR product in SW480, LoVo, and HCT116 cells but not in CCD-18Co cells. Unmethylated primers, on the contrary, were detected in CCD-18Co cells but not in SW480, LoVo, and HCT116 cells. The half-methylation primer failed to show clear differences among CCD-18Co, SW480, LoVo, and HCT116 cells (Fig. 4d–f). We quantitatively calculated the methylation level by dividing the methylation primer value or the half-methylation primer value by the unmethylated primer value. SW480, LoVo, and HCT116 cells showed significantly higher methylation levels than CCD-18Co cells when we used methylation primers but not when we used half-methylation primers (Fig. 4d–f). We next examined how sensitively the methylation primers could distinguish cancer cells from healthy cells in terms of the amount of template DNA. We observed the differential methylation levels of CCD-18Co and SW480 cells via qMSP and found that even 0.5 ng of template DNA, in the case of *PDX1*, was sufficient to observe the difference (Fig. 4g–i).

From these results, we confirmed that our MSP primers could distinguish cancer cells from normal cells very efficiently. Interestingly, although half-methylation primers also have four CpG sites where methylation levels between healthy and cancer cells are different, they could not produce clear differences when we executed MSP, suggesting that only MSP primers have more than enough CpG sites to provide substantially different results.

### The developed MSP primers could detect dynamic changes in methylation states

**Fig. 5:**
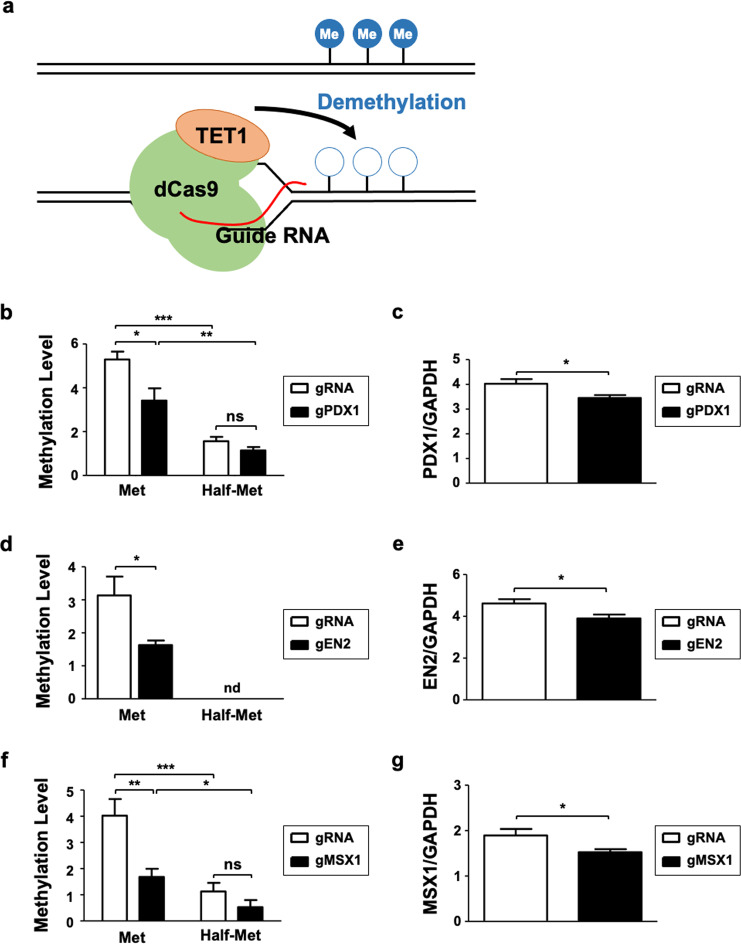
Customized MSP primers detect methylation changes in SW480 candidate biomarkers modulated by the CRISPR/dCas9-gRNA system. **a** A representation of our designed CRISPR/dCas9-gRNA system whereby specific gRNAs recruit the dCas9 protein and the catalytic domain of TET1 to demethylate the targeted genomic locus**. b**, **d**, **f** qMSP with SW480 cells transfected with dCas9-TET1CD mock or gRNA specific to **b** PDX1, **d** EN2, and **f** MSX1 indicates that the designed primers can distinguish the lack of methylation modulated by the CRISPR/dCas9-gRNA system compared with controls. **c**, **e**, **g** qPCR with SW480 cells transfected with dCas9-TET1CD mock or gRNA of **c** PDX1, **e** EN2, and **g** MSX1 shows a reduction in gene expression with decreased methylation. Genomic DNA and RNA used in qMSP and qPCR were simultaneously extracted from the cell lines.

We next examined whether our MSP primers could distinguish the dynamic changes in methylation levels out of concern that the data from cell lines might not sufficiently reflect physiological methylation changes due to fixed methylation values. To induce methylation changes, we used the CRISPR/dCas9-TET1 system (hereafter the dCas9-TET system), which enables us to decrease methylation levels in a location-specific manner (Fig. 5a)^45^. The gRNA targeting sites within 100 bp of the MSP primer binding site were searched and selected by Chopchopv2 and then the gRNA was subcloned into the dCas9-TET construct according to the predetermined process (Supplementary Fig. 9a-b).

After introducing the dCas9-TET system into the *PDX1* genomic region, confirmed by inspecting GFP expression (Supplementary Fig. 9c), we detected a significant reduction in methylation levels using our methylation primers, which contain seven CpG sites. However, we could not detect this difference using half-methylation primers (Fig. 5b). We noted that *PDX1* expression was significantly decreased according to the reduction in methylation level in the intragenic region, suggesting that the methylation changes are directly related to gene expression changes (Fig. 5c). We obtained similar results with *EN2* and *MSX1*. We successfully detected a reduction in the methylation levels in the intragenic regions of *EN2* and *MSX1* using our methylation primers, consistent with the reduction in gene expression (Fig. 5d–g). Thus, we concluded that our methylation primers are sensitive enough to detect methylation changes that precede gene expression changes.

### The methylation levels of *PDX1*, *EN2*, and *MSX1* predict CRC metastasis

**Fig. 6:**
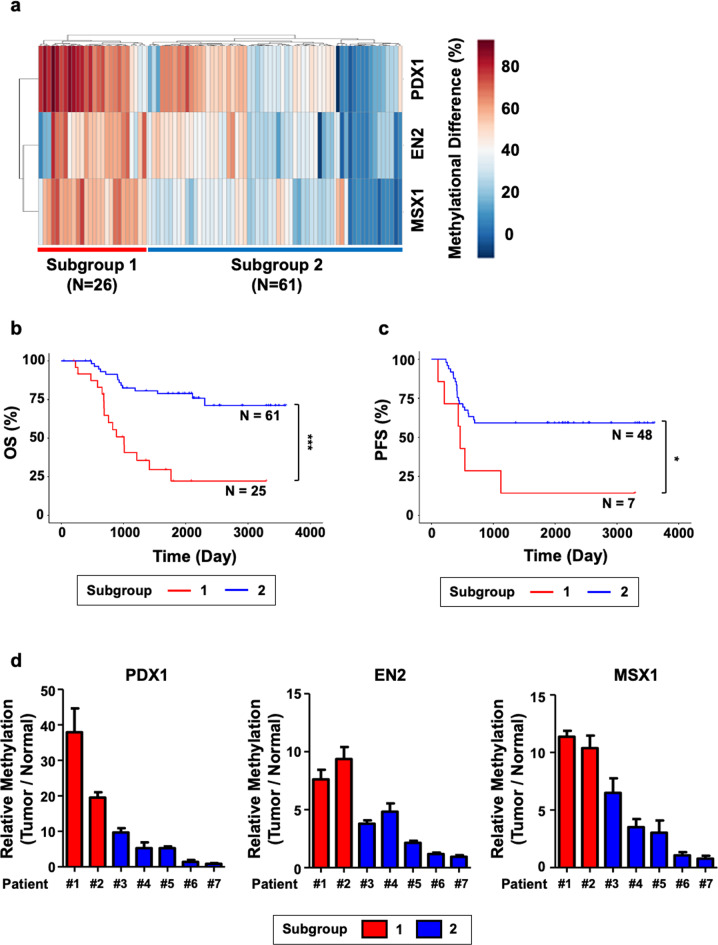
Prognostic potential of the 3-gene methylation signature is indicated through the classification of CRC patients. **a** Hierarchical clustering was conducted with DNA methylation data of intragenic CpG islands of *PDX1*, *EN2*, and *MSX1*, where two distinct subgroups of CRC patients were observed. **b**, **c** Kaplan–Meier plots for analyzing the significant differences in **b** overall survival and **c** CRC recurrence between the subgroups reveal the prognostic potential of the methylation data of the three biomarkers. The log-rank test was used to compare the significant differences between the two subgroups. One sample was excluded from the analysis of clinical data due to missing clinical data. Additionally, 31 patients were excluded from the recurrence analysis because they were diagnosed with stage IV CRC with metastatic cancers, and differentiating cancer recurrence would be challenging. **d** qMSP data generated with genomic DNA originating from the tumor and healthy tissues of the seven CRC patients displayed similar patterns to the cohort-specific methylation change analysis in **a**. The relative methylation levels of intragenic CpG islands of *PDX1*, *EN2*, and *MSX1* were calculated by dividing the methylation level of the tumor by that of healthy tissue.

Next, we examined whether the methylation levels of the intragenic CpG regions of *PDX1, EN2*, and *MSX1* have clinical implications. We classified patients based on the methylation levels of these regions by conducting hierarchical clustering with the Manhattan distance. Consequently, we created two groups: the hypermethylated group (Group 1, *N* = 26) and the intermediate methylation and hypomethylated group (Group 2, *n* = 61) (Fig. 6a). Interestingly, these two groups showed a substantial difference in OS (Fig. 6b) and PFS rates (Fig. 6c). In addition, peripheral lymphatic, vascular and perineural invasions, which are characteristic events followed by cancer metastasis, occurred more frequently in Group 1 than in Group 2. However, differences in cell differentiation, microsatellite instability, and tumor location were not observed. When we reviewed the information of our patients, we realized that the majority of stage IV (after metastasis) patients were included in Group 1, whereas the majority of stage III (before metastasis) patients were included in Group 2 (Table 2). These results suggest that *PDX1, EN2*, and *MSX1* methylation levels can predict CRC patient prognosis.

**Table 2:** Clinical data of the subgroups classified by the methylation level of the intragenic CpG island of PDX1, EN2, and MSX1; this data includes several patients, age, sex, cancer stage, invasion to adjacent tissues, cancer differentiation levels, microsatellite stability, and the origin of the tumor site.

Parameter	Subgroup 1	Subgroup 2	*P*
*N*	25	61	
Age, mean (range), year	58.2 (40–74)	63.2 (36–83)	0.0343*
Sex (male:female)	13:12	39:22	0.304, ns
Stage	*n* = 25	*n* = 61	
I	0% (0)	1.64% (1)	2.113E-06***
II	8% (2)	0% (0)	
III	20% (5)	78.7% (48)	
IV	72% (18)	26.2% (12)	
Invasion	*n* = 25	*n* = 61	
Lymphatic	56% (14)	45.9% (19)	0.0314*
Vascular	44% (11)	19.6% (8)	0.00172**
Perineural	80% (20)	50.8% (31)	0.0124*
Differentiation	*n* = 24	*n* = 58	
Well	0% (0)	1.7% (1)	0.706, ns
Moderate	91.7% (22)	93.1% (54)	
Poor	8.3% (2)	5.2% (3)	
Microsatellite	*n* = 23	*n* = 58	
Stable	91.3% (21)	93.1% (54)	0.969, ns
Instable - Low	4.3% (1)	5.2% (3)	
Instable - High	4.3% (1)	5.2% (3)	
Site of Tumor	*n* = 25	*n* = 58	
Ascending	20% (5)	25.9% (15)	0.667, ns
Descending	4% (1)	0% (0)	
Transverse	4% (1)	1.7% (1)	
Sigmoid	40% (10)	36.2% (21)	
Rectal	16% (4)	20.7% (12)	
Rectosigmoid Junction	16% (4)	15.5% (9)	

The age of the two subgroups was compared via a two-tailed *t* test, and the chi-square test was used to analyze the other parameters. Significance levels are presented as ns: nonsignificant, *p < 0.05, **p < 0.01, ***p < 0.001.

Finally, we examined whether our MSP system could distinguish between these two patient groups. We executed qMSP using bisulfite-treated genomic DNA from the tumor tissues of seven patients. Two patients in Group 1 showed higher methylation levels in the intragenic regions of *PDX1, EN2*, and *MSX1* than five individual patients in Group 2 (Fig. 6d). This result suggests that our MSP detection system can be clinically applied to predict the prognosis and metastasis of CRC patients after surgery.

## Discussion

In this study, we present our discovery of novel CRC prognostic markers based on a comprehensive analysis of multiomics data and the validation of their functional impact in vitro. First, we used a public database for the preliminary screening of CRC-specific differentially methylated regions. In addition, we generated high-coverage targeted bisulfite sequencing data from the South Korean CRC cohort. For functional validation, we analyzed RNA-seq data and generated CRISPR/dCas-based cell lines. Finally, we established qMSP-based primer sequences and protocols for the quick and easy prediction of CRC prognosis.

We aimed to identify intragenic CGIs in which methylation changes were significantly related to gene expression and further cancer progression. By examining the differences in the methylation levels observed in tumors and adjacent healthy tissues via hybrid capture-based targeted bisulfite sequencing, we discovered significantly hypermethylated intragenic CGI regions in *PDX1, EN2*, and *MSX1* in the tumor samples. Therefore, we selected genomic locations targeted by MSP and designed primers to validate the hypermethylated status of the target CGIs. Our primer design system for the candidate methylation biomarkers provided the strength that enabled the effective detection of methylation changes. In other words, since the targeted bisulfite sequencing data showed the methylation level of almost all CpG sites in certain genomic regions of interest, we could select the optimal MSP target sites efficiently, where the differences in methylation levels between healthy and tumor tissues were significant (Fig. 4a–c and Supplementary Fig. 8). Hence, we successfully identified tumor-specific differentially methylated CGIs as prognostic markers of CRC and developed optimized qMSP methods to detect these methylation markers effectively.

Despite extensive efforts to discover CRC prognostic markers, technical drawbacks have challenged many researchers in developing systems for the clinical application of these biomarkers. One of the most important reasons is the difficulty in optimizing the qMSP. Specifically, the methylation level is difficult to quantify when discriminating between bisulfite-treated cytosine (methylated C) and uracil (unmethylated C) simultaneously. Increasing primer sensitivity while removing nonspecific bands is the key hurdle for optimizing qMSP. Based on high-coverage targeted bisulfite sequencing data, we identified well-performing primer sets that included six or seven CpG sites in the forward and reverse primers that significantly distinguished healthy tissues from tumor tissues, although these primer sets were not tested in a multiplexing mode of action. We assume that more CpG sites can increase the annealing temperature, which could be more effective in precisely binding primers to their target sites. The qMSP technique established in this study may be used in additional and more feasible clinical applications for prognosis prediction if it is further developed and optimized as a multiplex qMSP technique.

After inspecting the DNA methylation levels of the genes of interest, we then investigated the correlation between epigenetic regulation and the subsequent gene expression changes that ultimately lead to DNA methylation. However, even if there are significant epigenetic changes, one cannot conclude that these changes are correlated with gene expression. For example, we found two CpG islands of the *HOXA3* gene as the top 1 (chr7:27,147,589-27,148,389; hereafter *HOXA3*_CGI 7) and 2 (chr7:27,146,069-27,146,600; hereafter *HOXA3*_CGI 6) candidates that satisfied our criteria, but we failed to determine whether the expression of *HOXA3* was significantly changed in CRC patients (Table 1, Supplementary Figs. 5 and 10). We suppose that even if it is technically possible to detect the methylation changes of a particular gene of interest, it is still not a suitable epigenetic marker unless there is confidence in its expression effects.

While it is well known that hypermethylation of promoter CpG islands leads to decreased gene expression, the mechanism and regulatory roles with respect to the gene expression of hypermethylated intragenic CGIs are still debated^19,36^. One of the arguments supporting the idea of tumorigenesis caused by the hypermethylation of intragenic CGIs is that it leads to the hypermethylation of certain homeobox genes in their gene body^46^. This phenomenon was also confirmed in our study because *PDX1, EN2*, and *MSX1* are members of the homeobox family of genes. In addition to the *PDX1, EN2*, and *MSX1* CGIs, several CGI regions in other genes are worth examining. Many researchers have found methylated biomarkers in *BCAT1, NDRG4, SEPT9, BMP3*, and *IKZF1*^47–50^, which correlates with our findings (Supplementary Figs. 11–15). Therefore, we provide evidence supporting the role of intragenic CGIs, which warrants further research.

In this study, we propose a practical method for identifying CRC prognostic markers. We utilized public databases and generated suitable high-depth targeted bisulfite sequencing data to define South East Korean-specific differentially methylated regions (DMRs). We also validated the proliferative aspect of the intragenic CGIs of *PDX1, EN2*, and *MSX1* in vitro, and we present optimized qMSP methods for further application in clinical fields. Based on the follow-up data of the patients in the cohort, we found a significant decrease in OS and higher recurrence rates in CRC patients with hypermethylated target CGIs. Along with surgical biopsy, adjuvant chemotherapy, and other proper care, regular tracking of prognostic factors could be helpful for patients with late-stage CRC. We also expect that our proposed methods and biomarkers could be applied to other cancers.

## Supplementary information

Supplementary Information

## Data Availability

All data generated and used in this study are available for anyone to use without violating participant confidentiality. Additional information can be requested from the corresponding author for the appropriate reasons.
