# Supplementary material for: Hypermethylation of PDX1, EN2, and MSX1 predicts the prognosis of colorectal cancer
Source: Exp Mol Med. 2022 Feb 15;54(2):156–68. doi: 10.1038/s12276-022-00731-1 (PMC8894425; doi:10.1038/s12276-022-00731-1)
Supplement: Supplementary file 1 — Supplementary Information [file 12276_2022_731_MOESM1_ESM.pdf]

Supplementary Materials and Methods

Analysis of TCGA colon adenocarcinoma RNA sequencing data

Three hundred and twenty read count files (healthy tissue sample=41, tumor sample=279) which had been quantified with HTSeq, were used to analyze the CRC gene expression pattern of healthy tissue and tumor samples. Each read count was integrated into a matrix format, and the list of differentially expressed genes between healthy tissue and tumors was generated using the DeSeq2 package (version 3.12) in R software. Meanwhile, the TPM value of each gene was derived by using the scaled-estimate value from TCGA RNA-seq V2 data and multiplying by 10^6. Genes that showed a greater than 2-fold change in both count and TPM data with statistical significance (adjusted p-value < 0.05) between normal and tumor samples were shortlisted.

The overexpression construct

Each overexpression construct of the candidate genes was subcloned from the pcDNA3-N-Flag-NLRP3 vector. To obtain insert fragments, we designed PCR primers that specifically amplified target sequences on HCT116 and SW480 cDNA with reference for the coding sequences of the candidate genes in the National Center for Biotechnology Information. As the target genes have numerous CpG sites, the melting temperature (Tm) of the target amplicon naturally increases, hindering the PCR reaction. Thus, we pre-boiled HCT116 and SW480 cDNA for 10 min before commencing PCR to completely separate the double-stranded structure of the template DNA.

Cell cultures

We used the colon cancer cell lines HCT116, LoVo, SW480, and a healthy colon fibroblast cell line, CCD-18Co. CCD-18Co was purchased from the Korean Cell Line Bank (KCLB), and HCT116, LoVo, SV480 cell lines were kindly gifted by Prof. Sungsoon Fang of Yonsei University South Korea. HCT116, LoVo, and SW480 cells were maintained in RPMI 1640 medium (11875119; Gibco) with 10% fetal bovine serum (Characterized Australian Fetal Bovine Serum, SH30084.03; Hyclone), and CCD-18Co was grown in DMEM (DMEM/High glucose with L-glutamine, sodium pyruvate with phenol Red, SH30243.01; Hyclone) with

10% FBS. All cell lines were incubated at 37 °C and 5% CO_2 in a humidified incubator. For overexpression of candidate genes *in vitro*, HCT116 cells were seeded in 60-mm culture plates and transfected with either an empty vector or a construct with the candidate genes using Lipofectamine 2000 (11668019; Thermo Fisher Scientific, Massachusetts, USA). The transfection efficiency of each overexpression construct was confirmed by the western blotting of the tags. SW480 cells were transfected with the dCas9-TET1 construct using Lipofectamine3000 (L3000015; Thermo Fisher Scientific) to enhance transfection efficiency. Transfection efficiencies were verified using GFP as detected by fluorescence microscopy (Cell Imaging System, fl_AMF-4306; EVOS). To detect DNA methylation status and mRNA expression level simultaneously, both genomic DNA and total RNA were extracted from a single sample (AllPrep DNA/RNA mini kit, 80204; Qiagen) and subjected to qMSP and qPCR, respectively.

Western blotting

Western blotting was conducted to confirm the overexpression of candidate genes compared with the empty vector by immunoblotting FLAG-tags at the N-terminus of each construct. Whole cell extracts were isolated using RIPA buffer (9806S; Cell Signaling Technology) supplemented with protease inhibitors (QTPPI1015; Bio-Rad). The concentration of the isolated proteins was determined using BCA Protein Assay Reagent (23209, 23224, 23228; Thermo Fisher Scientific). Fifteen-twenty micrograms of the protein were separated on a 3-8% Tris-acetate gel and transferred to PVDF membranes (Millipore, Billerica, MA). Membranes were then incubated with the primary antibodies against the FLAG epitope (Sigma, St. Louis, MO) or -actin (Abcam, Cambridge, MA) and the appropriate secondary antibodies.

Cell proliferation assay

A total of 1×10^5 HCT116 cells were transfected with the gene construct for 24 h, followed by seeding in 24-well plates. 40ul of Cell Counting kit-8 (CK04-11; Dojindo, Kumamoto, Japan) solution was added to 400ul culture media of each well and then incubated for 1 hour. Cell viability was determined by measuring the absorbance at 450 nm using a microplate reader (Molecular Devices, LLC) at 450 nm at the indicated time points.

Invasion assay

Invasion assays were performed in 24-well transwell plates (8-µm pore size, 3422; Costar). For invasion assays, 2×10^5 HCT116 cells were transfected with the gene construct for 24 h, followed by seeding in Matrigel-coated upper chambers. The upper chamber was filled with serum-free RPMI medium, while the lower chamber was filled with RPMI medium supplemented with serum as a chemoattractant. After incubation for 48 h, the cells that had not invaded through the membrane were removed, and the invaded cells were stained with crystal violet and counted.

MSP primer design

To validate DNA hypermethylation in candidate CpG islands *in vitro*, we used the following criteria to design MSP primers. First, the Tm difference between the forward and reverse primers was less than 2 °C. Tm, which was calculated using Oligo Calc (version 3.27), was set between 55 °C and 60 °C. Primer length was designated as 22 bp to 33 bp, with the expected PCR amplicon size set between 100 bp and 160 bp [25]. Additionally, with reference to the DNA methylation status in the generated targeted bisulfite sequencing data, we designed MSP primers to include at least six CpG sites in the primer binding regions. Finally, regions where more than 2/3 of the CpG sites are methylated by less than 20% in healthy tissues, and more than 5/6 are methylated by more than 50% in tumors were selected for primer binding. MSP primer sets that bind to methylated (Met), or unmethylated CpG sites (Unmet) were designed manually using our criteria. Additionally, we also included primers that bind to partially methylated CpG sites (Half-Met).

Quantitative methylation-specific PCR (qMSP)

Prior to measuring DNA methylation levels of target genes, 500 ng of genomic DNA extracted from colorectal cell lines or CRC patients was treated with sodium bisulfite (EZ DNA Methylation-Lightning Kits, D5031; Zymo Research). Concentration of bisulfite-converted genomic DNA was quantified using a UV spectrophotometer (Nanodrop 2000; Thermo Fisher Scientific). In the qMSP reaction, we used the qPCR master mix (KAPA

SYBR FAST qPCR Master Mix (2X), KK4608; Kapa Biosystems), which enhanced the GC-rich PCR with a PCR cycler (LightCycler 480 II; Roche Diagnostics). The crossing point (Cp) value was calculated by directly adjusting the signal threshold. The DNA methylation level of each CpG island was calculated using the following equation:

$$\text{(Methylation level)} = 2^{\text{(Cp of Unmet)} - \text{(Cp of Met)}}.$$

The CRISPR/dCas9-TET1 construct with guide RNA (gRNA)

gRNA targeting sites within 100 bp of the MSP primer binding site were selected through Chopchopv2 and further filtered for the least number of off-target sites and best targeting efficiency (Labun et al., 2016). The cloning process was conducted according to the gRNA cloning protocol of Mali P (Mali et al., 2013; Morita et al., 2016). The Gibson ligation step was performed using an In-Fusion cloning kit (639649; Takara Bio Inc., Shiga, Japan), and the cloned gRNA sequence was confirmed by pyrosequencing.

Quantitative PCR (qPCR)

To check the expression of each candidate gene upon demethylation via the dCas9 system, we synthesized complementary DNA from the total RNA using reverse transcriptase (18090050; Invitrogen). qPCR was performed as per qMSP.

Kaplan–Meier survival estimation

To investigate patient survival according to the expression level of the candidate genes, we utilized the UALCAN database (http://ualcan.path.uab.edu/index.html). Genes of interest were tabulated in a specified format, and the appropriate cancer type for analysis was pre-selected. UALCAN results culminated in the categorization of two groups: (1) high expression of queried genes (upper 25%) and (2) low/medium expression of queried genes (lower 75%). To evaluate the prognostic potential of methylation in the intragenic regions of *PDX1*, *EN2*, and *MSX1* in CRC, we used the survival (version 3.2-7), survminer (version 0.4.8), and ggplot2 packages (version 3.3.3) in R software. Progression-free survival in cancer recurrence analysis and overall survival (OS) in the survival analysis of CRC patients were

evaluated. The statistical significance of the survival ratio was calculated using the log-rank test.

Supplementary Figures

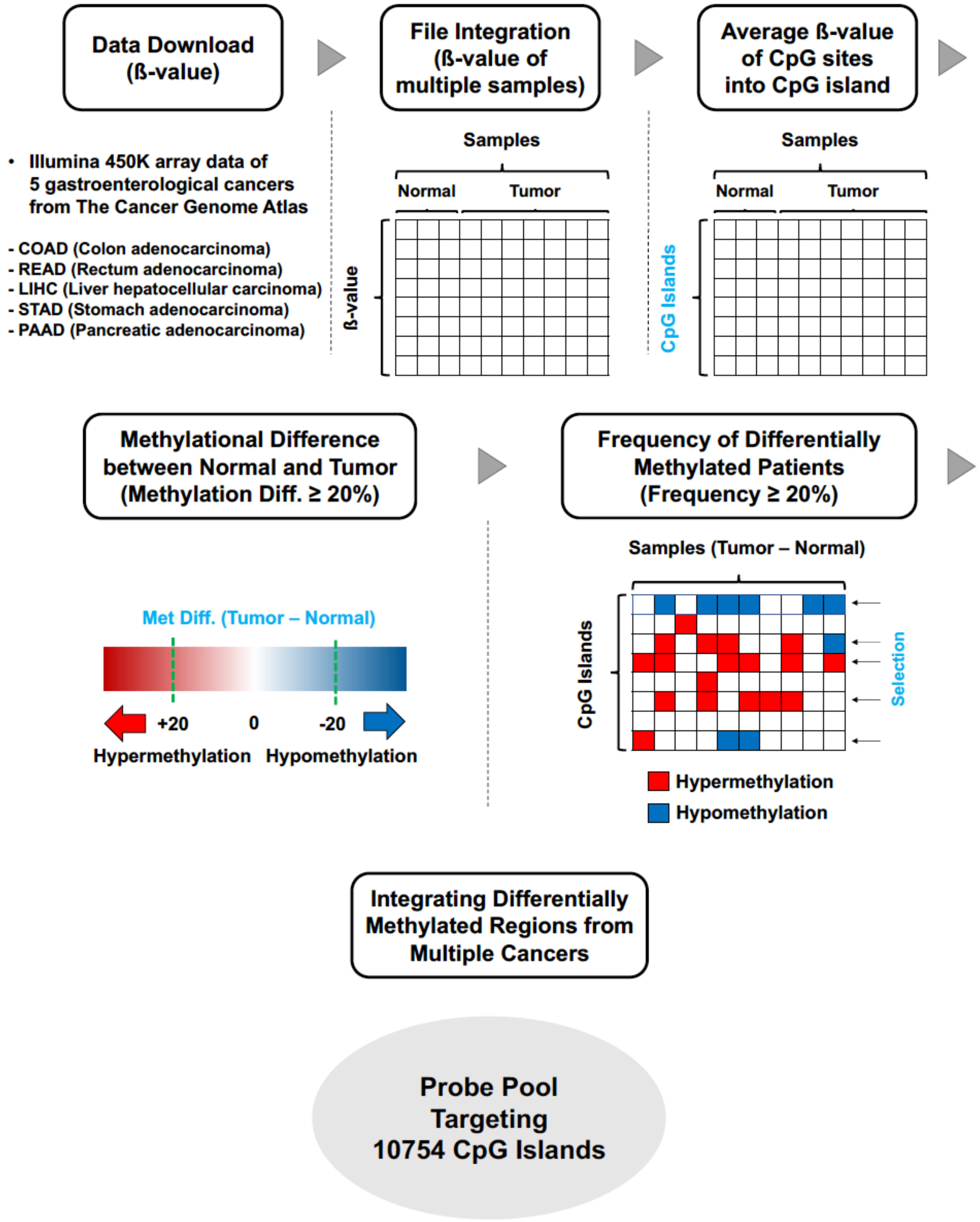

Supplementary Fig. 1 TCGA Illumina 450K Array Data Preprocessing.

The Illumina Infinium 450 K microarray data from The Cancer Genome Atlas were downloaded from the GDC data portal of the National Institutes of Health (NIH). Each sample

contained a beta value of approximately 450,000 probes. To estimate the methylation value of CpG islands, CpG sites on the same CpG island according to hg19 were averaged in each array datum. Differences in methylation values between tumors and an average of healthy tissues were calculated, and CpG islands in which methylation levels differed by > 20% between tumors and the average of healthy tissues in 20% or more of the total patients were selected. A total of 10,754 CpG islands showed differential methylation, and the resulting list was used to target the probe pool design.

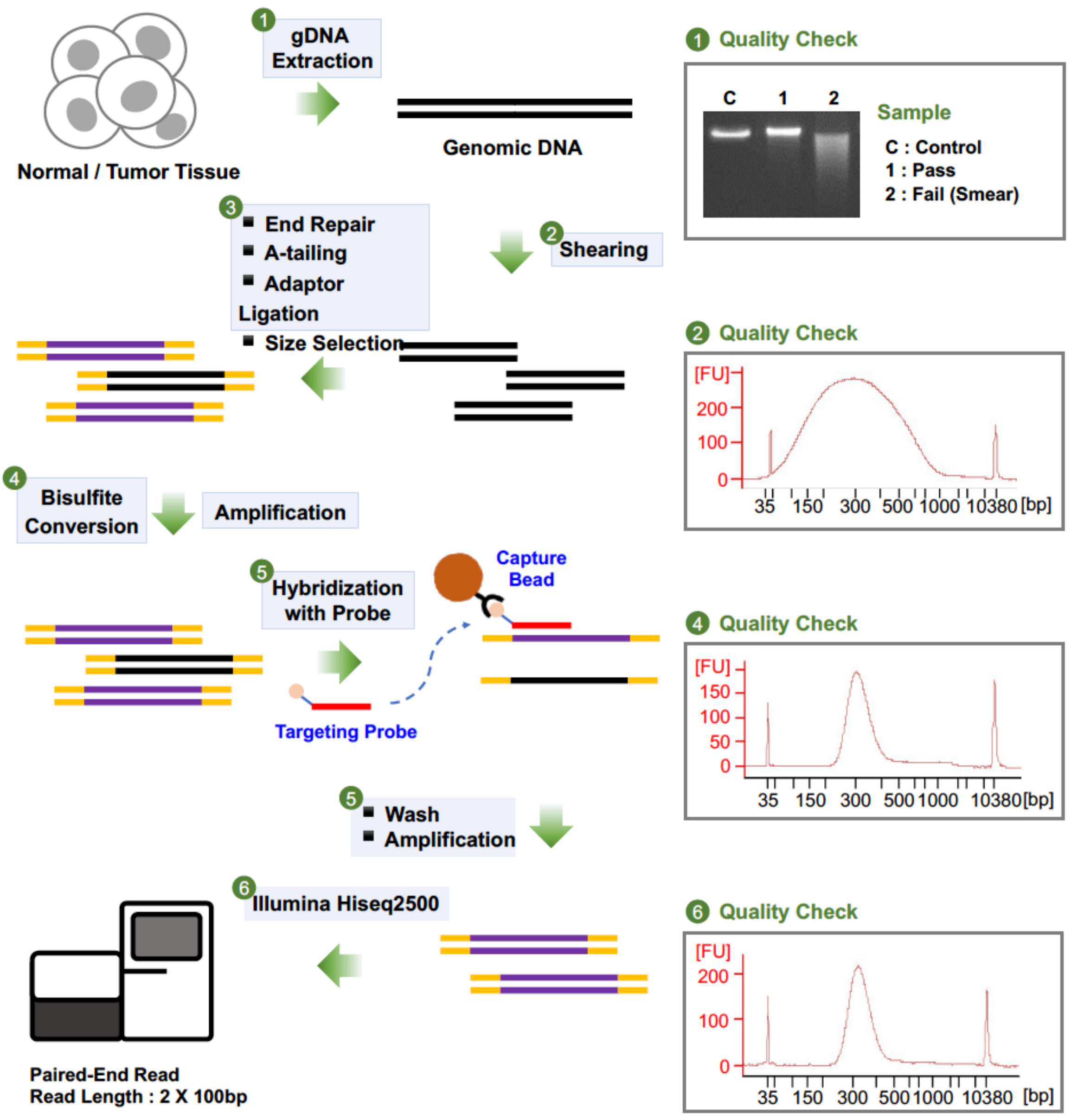

Supplementary Fig. 2 Preparation of the targeted DNA methylation sequencing library.

Genomic DNA from healthy and tumor tissues from the CRC cohort was extracted. Only QC-passed samples were used for the preparation of the targeted bisulfite sequencing library. Each genomic DNA was sheared to 250-300 bp, the gold standard for high-throughput sequencing. Single-stranded ends of sheared genomic DNA were repaired, followed by A-tailing, adaptor ligation, and size selection. Bisulfite conversion of genomic DNA was conducted to differentiate unmethylated cytosines from their methylated counterparts. To recover an appropriate quantity of bisulfite-converted genomic DNA, PCR amplification was performed before and after hybridization. After each amplification step, the quality and quantity of the

PCR products were confirmed using the Agilent 2100 Bioanalyzer system. The prepped samples were then used for high-throughput sequencing using Hiseq2500.

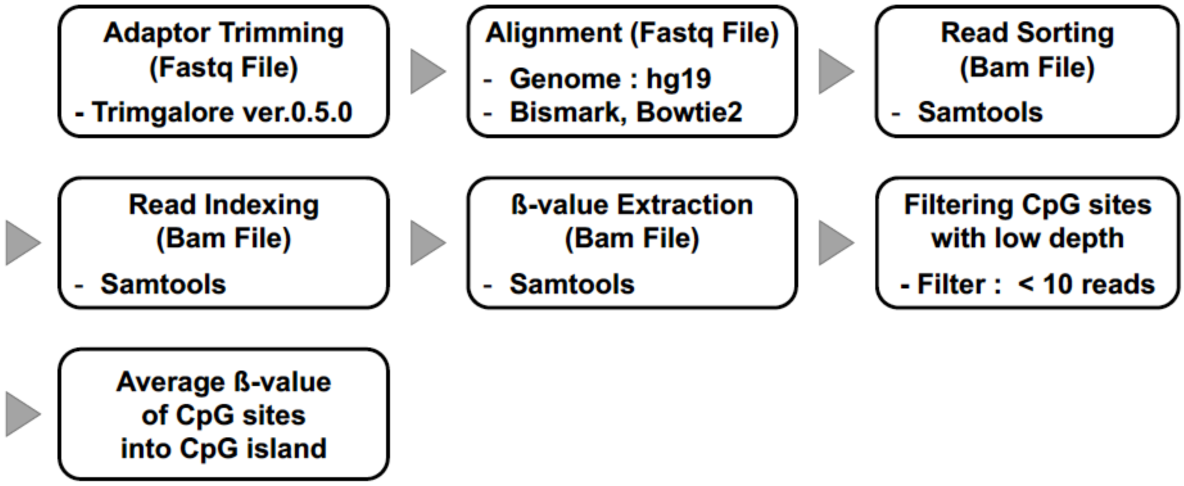

Supplementary Fig. 3 Targeted bisulfite sequencing data pre-processing.

Trimgalore (ver. 0.5.0) was used to trim the adaptor sequence from each targeted bisulfite sequencing data, and sequencing reads were aligned on the hg19 human genome reference using Bismark and Bowtie2. The sequencing reads were then sorted and indexed, and their methylation counts were extracted. CpG sites with a read depth below 10 were filtered out. Methylation values of CpG sites were averaged to estimate the methylation values of CpG islands.

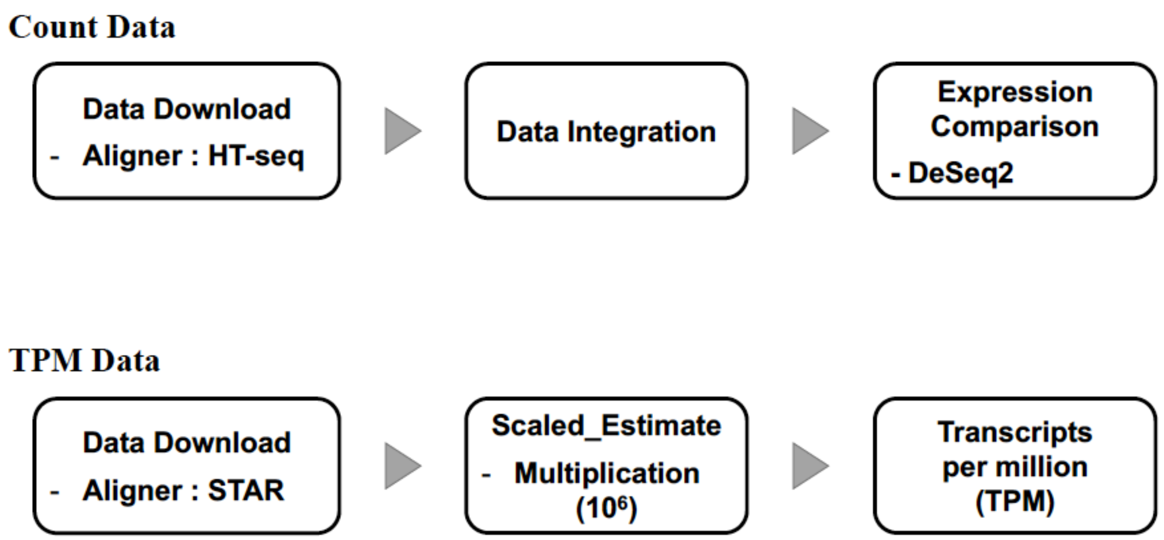

Supplementary Fig. 4 TCGA RNA-seq data pre-processing.

Count data aligned by HT-seq were downloaded. Each RNA-seq data was integrated into a matrix, and gene expression differences between tumor and healthy tissues were calculated using DESeq2. To obtain normalized gene expression data (TPM value), the scaled-estimate value of RNA-seq data aligned by STAR was multiplied by 10^6.

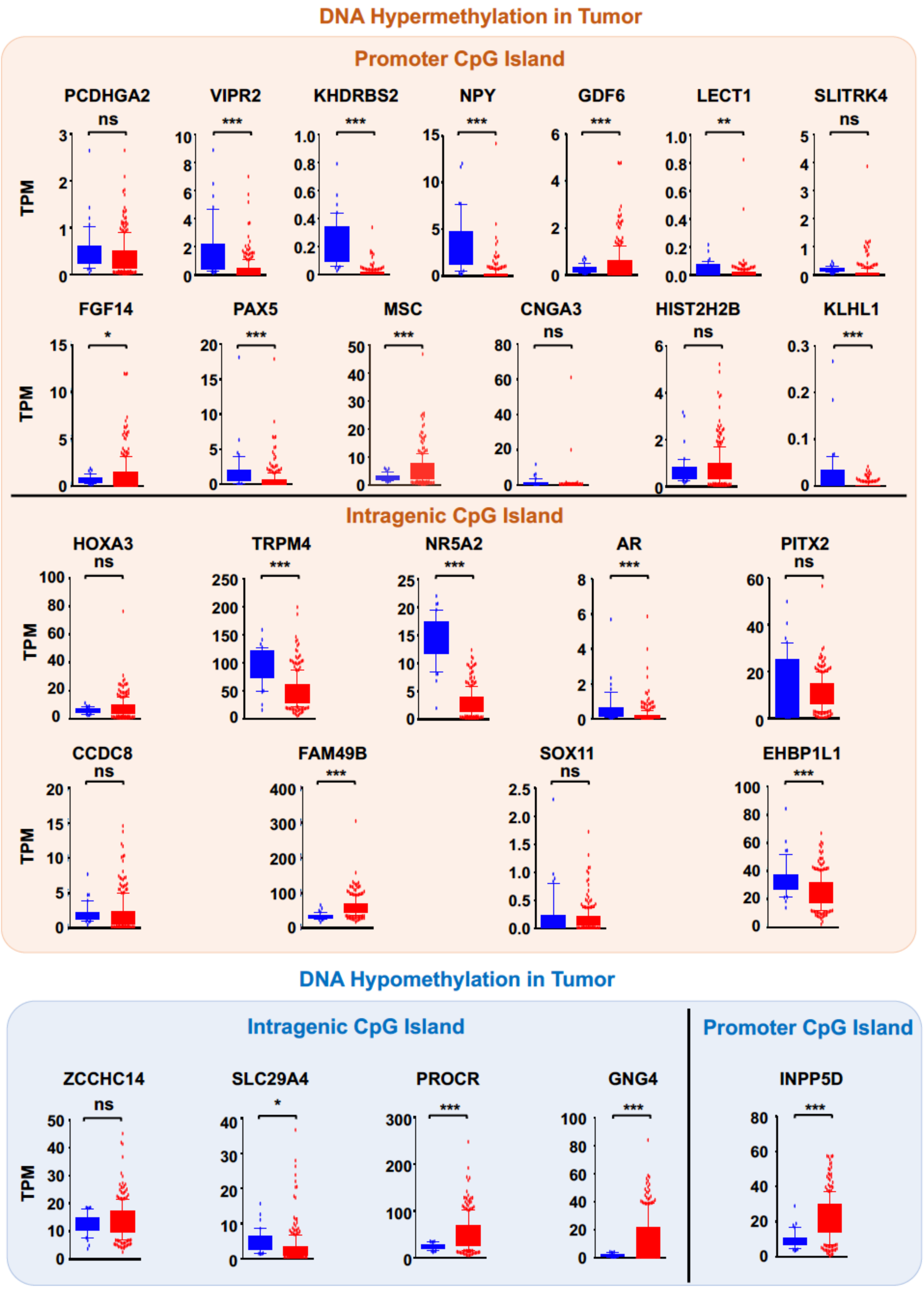

Supplementary Fig. 5 Expression levels of candidate methylation biomarker genes.

The TPM values of the genes from Table 1 are listed. RNA-seq data of candidate genes from healthy and tumor colon tissues were downloaded from TCGA, and TPM values were calculated by multiplying the scaled-estimate value of RNA-seq data by 10^6.

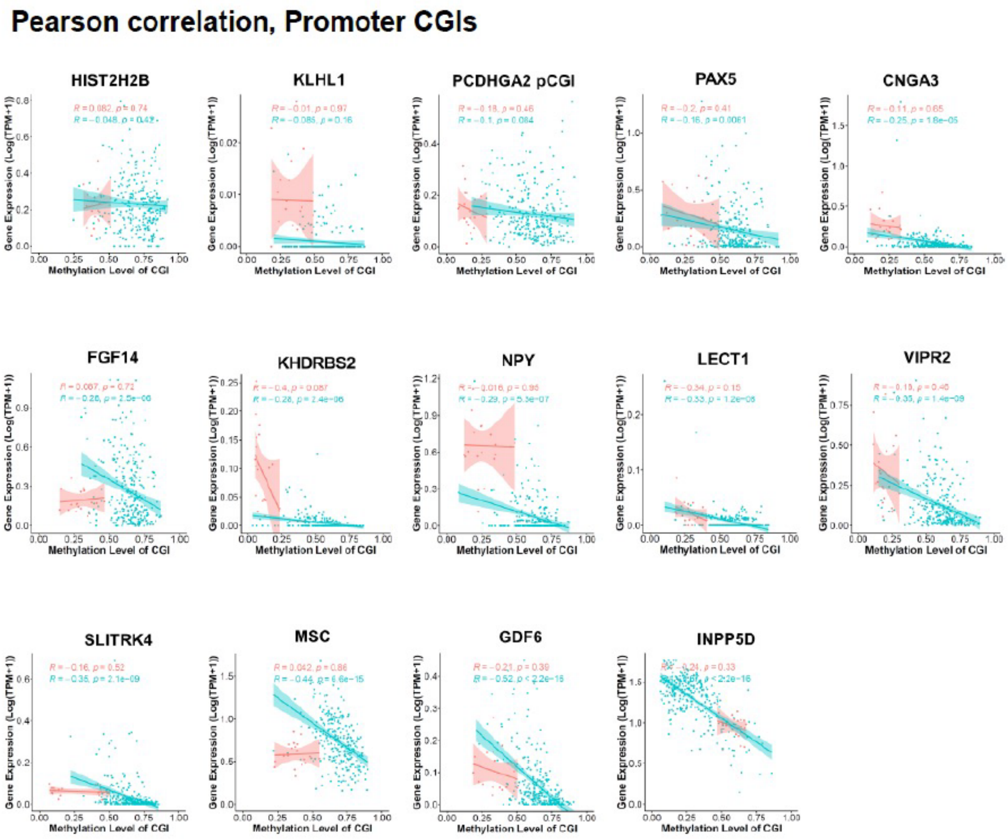

CGI location	Methylation	CpG island	Pearson	p value	Spearman	p value
Promoter	Hyper	HIST2H2BA	-0.048	0.42	-0.064	0.29
		KLHL1	-0.085	0.16	-0.051	0.4
		PCDHGA2	-0.1	0.084	-0.15	**0.012**
		PAX5	-0.16	**0.0061**	-0.22	**$2.6X10^{4}$**
		CNGA3	-0.25	**$1.8X10^{5}$**	-0.32	**$3.6X10^{8}$**
		FGF14	-0.28	**$2.5X10^{6}$**	-0.26	**$1.3X10^{5}$**
		KHDRBS2	-0.28	**$2.4X10^{6}$**	-0.37	**$1.9X10^{10}$**
		NPY	-0.29	**$5.3X10^{7}$**	-0.22	**$2.5X10^{4}$**
		LECT1	-0.33	**$1.2X10^{8}$**	-0.16	**$8.7X10^{3}$**
		VIPR2	-0.35	**$1.4X10^{9}$**	-0.38	**$9.4X10^{11}$**
		SLITRK4	-0.35	**$2.1X10^{9}$**	-0.46	**$3.8X10^{16}$**
		MSC	-0.44	**$6.6X10^{15}$**	-0.48	**$<2.2X10^{16}$**
		GDF6	-0.52	**$<2.2X10^{16}$**	-0.48	**$<2.2X10^{16}$**
	Hypo	INPP5D	-0.67	**$<2.2X10^{16}$**	-0.54	**$<2.2X10^{16}$**

Supplementary Fig. 6 Pearson correlation between promoter CGI methylation and matched gene expression

To find the correlation between CGI methylation level and gene expression of candidate promoter CGIs, we called the Infinium humanmethylation450 data and RNA sequencing from TCGA-COAD (Normal = 19, Tumor = 279). RNA-Seq TPM data were log-transformed to reduce and correct the difference between Pearson and Spearman correlation coefficient

[Log(TPM+1)]. Red indicates normal and blue indicates tumor. The correlation coefficient of tumor samples is organized in a table.

Pearson correlation, Intragenic CGIs

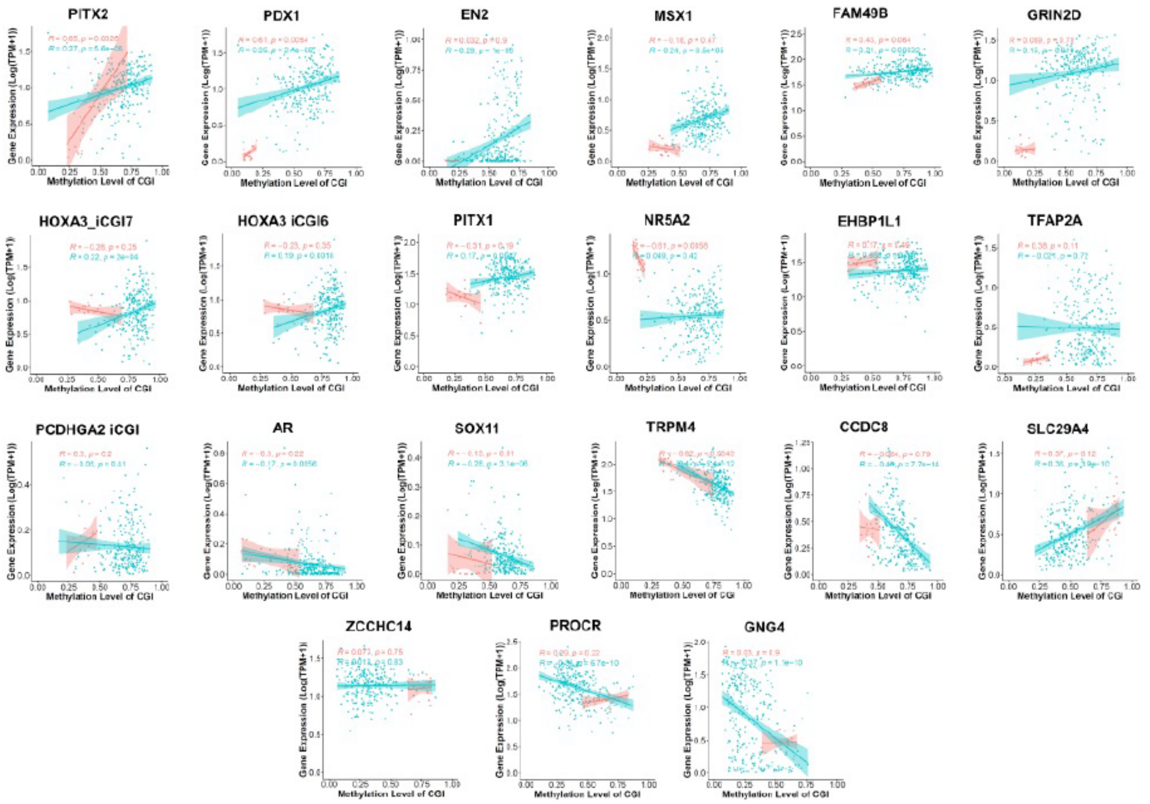

CGI location	Methylation	CpG island	Pearson	p value	Spearman	p value
Intragenic	Hyper	EN2	0.29	**$1X10^{6}$**	0.25	**$2.9X10^{5}$**
		PITX2	0.27	**$5.6X10^{6}$**	0.3	**$5.5X10^{7}$**
		PDX1	0.25	**$2.4X10^{5}$**	0.28	**$1.6X10^{6}$**
		MSX1	0.24	**$6.5X10^{5}$**	0.23	**$8X10^{5}$**
		HOXA3 (iCGI7)	0.22	**$2X10^{4}$**	0.18	**0.0027**
		FAM49B	0.21	**0.00032**	0.19	**0.0014**
		HOXA3 (iCGI6)	0.19	**0.0018**	0.13	**0.031**
		PITX1	0.17	**0.0047**	0.1	0.091
		GRIN2D	0.15	**0.014**	0.18	**0.0025**
		EHBP1L1	0.088	0.14	0.042	0.48
		NR5A2	0.049	0.42	0.063	0.17
		TFAP2A	-0.021	0.72	-0.04	0.51
		PCDHGA2	-0.05	0.41	-0.019	0.76
		AR	-0.17	**0.0056**	-0.2	**0.00076**
		SOX11	-0.28	**$3.1X10^{6}$**	-0.27	**$3.2X10^{6}$**
		TRPM4	-0.4	**$7.4X10^{10}$**	-0.36	**$7.4X10^{10}$**
		CCDC8	-0.43	**$7.4X10^{10}$**	-0.46	**$<2.2X10^{16}$**
	Hypo	SLC29A4	0.36	**$3.9X10^{10}$**	0.33	**$1.6X10^{8}$**
		ZCCHC14	0.013	0.83	0.026	0.66
		PROCR	-0.36	**$6.7X10^{10}$**	-0.35	**$2.1X10^{9}$**
		GNG4	-0.37	**$1.1X10^{10}$**	-0.35	**$1.7X10^{9}$**

Supplementary Fig. 7 Pearson correlation between intragenic CGI methylation and matched gene expression

To find the correlation between CGI methylation level and gene expression of candidate intragenic CGIs, we called the Infinium humanmethylation450 data and RNA sequencing from TCGA-COAD (Normal = 19, Tumor = 279). RNA-Seq TPM data were log-transformed to reduce and correct the difference between Pearson and Spearman correlation coefficient [Log(TPM+1)]. Red indicates normal and blue indicates tumor. The correlation coefficient of tumor samples is organized in a table.

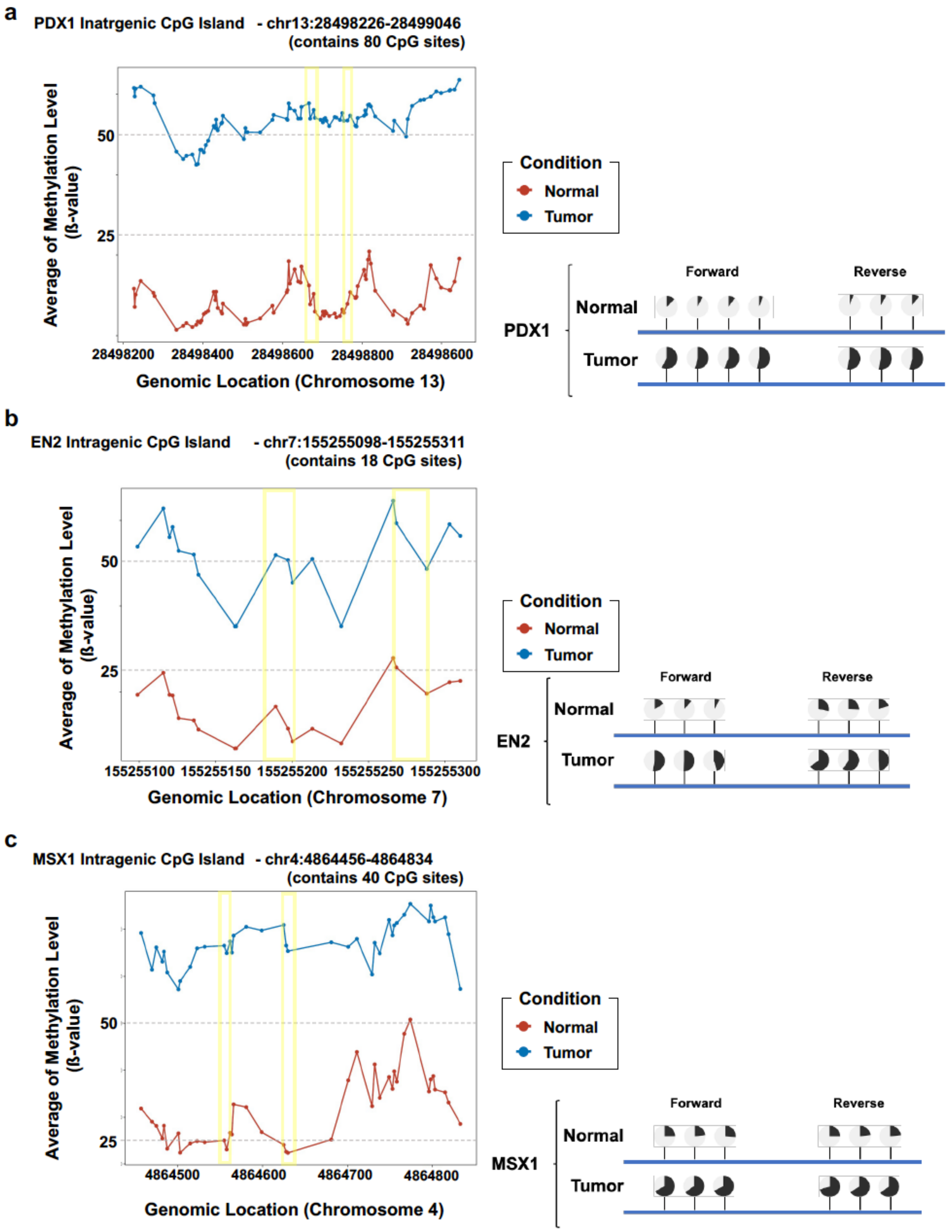

Supplementary Fig. 8 MSP targeting genomic regions in the intragenic CpG island of *PDX1*, *EN2,* and *MSX1*.

a-c) Line graph indicating the average DNA methylation level of the CpG sites in the candidate CpG island and their targeted MSP primer binding sites. Targeted bisulfite

sequencing data were used in the plotting process. **(Left)** The red line represents the average methylation level of healthy samples, while the blue line corresponds to tumor samples. Each dot in the line graph denotes the CpG sites included in the CpG island. The yellow boxes indicate the MSP forward and reverse primer binding sites. **(Right)** DNA methylation status of CpG sites in healthy and tumor colon tissues where custom-made MSP primers anneal. Each dot represents the CpG site, and the dark portion of each dot represents the average methylation level.

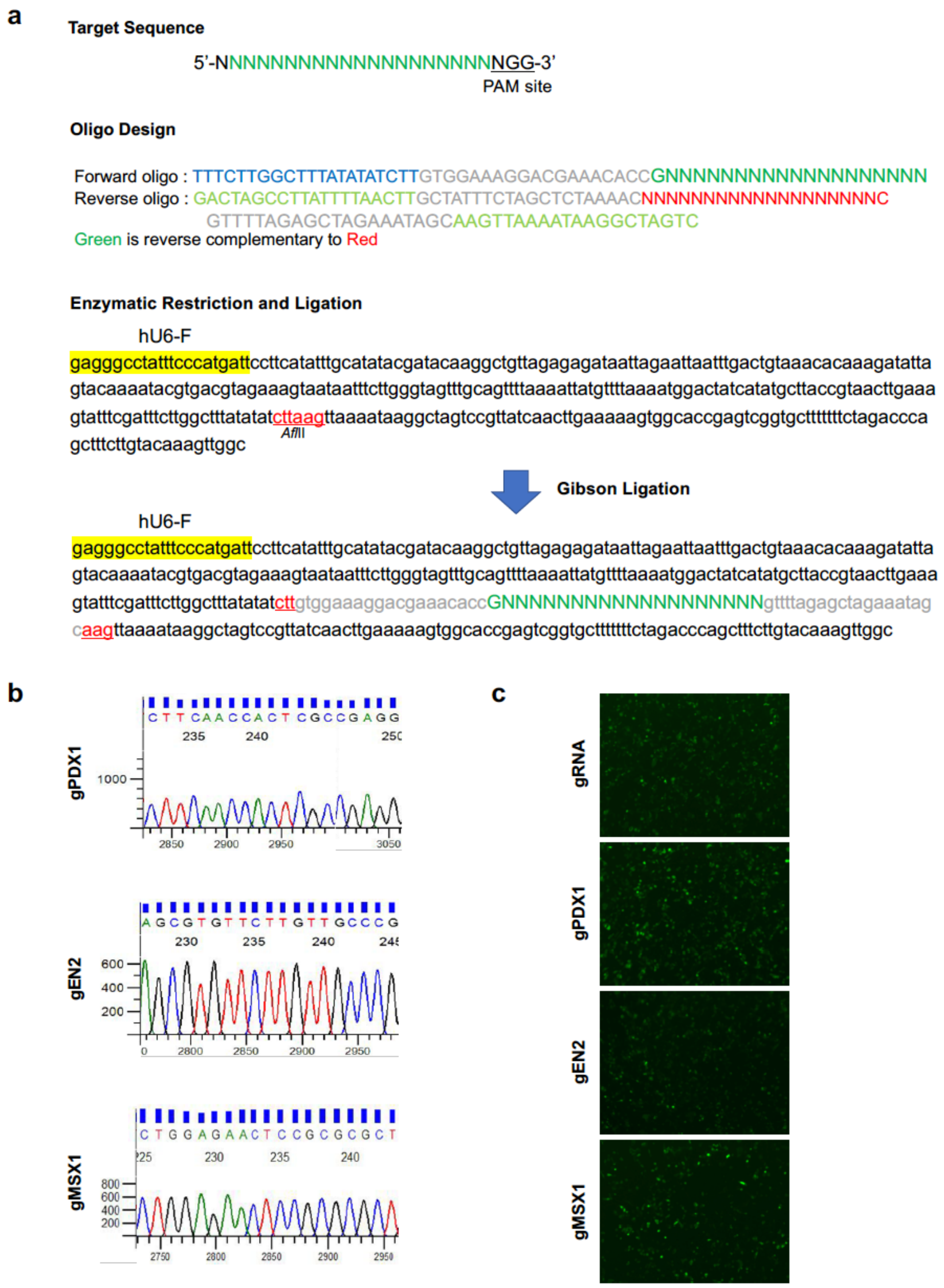

Supplementary Fig. 9 Subcloning of pPlatTET-gRNA2.

a) The design scheme of subcloning gRNA in the dCas9-TET1CD vector. **b)** Pyrosequencing results of subcloned vectors are shown, and each gRNA coding sequence was validated through manual inspection. **c)** GFP expression in SW480 cells transfected with dCas9-TET1CD vectors. The GFP expression levels in each transfection trial were verified in each experiment.

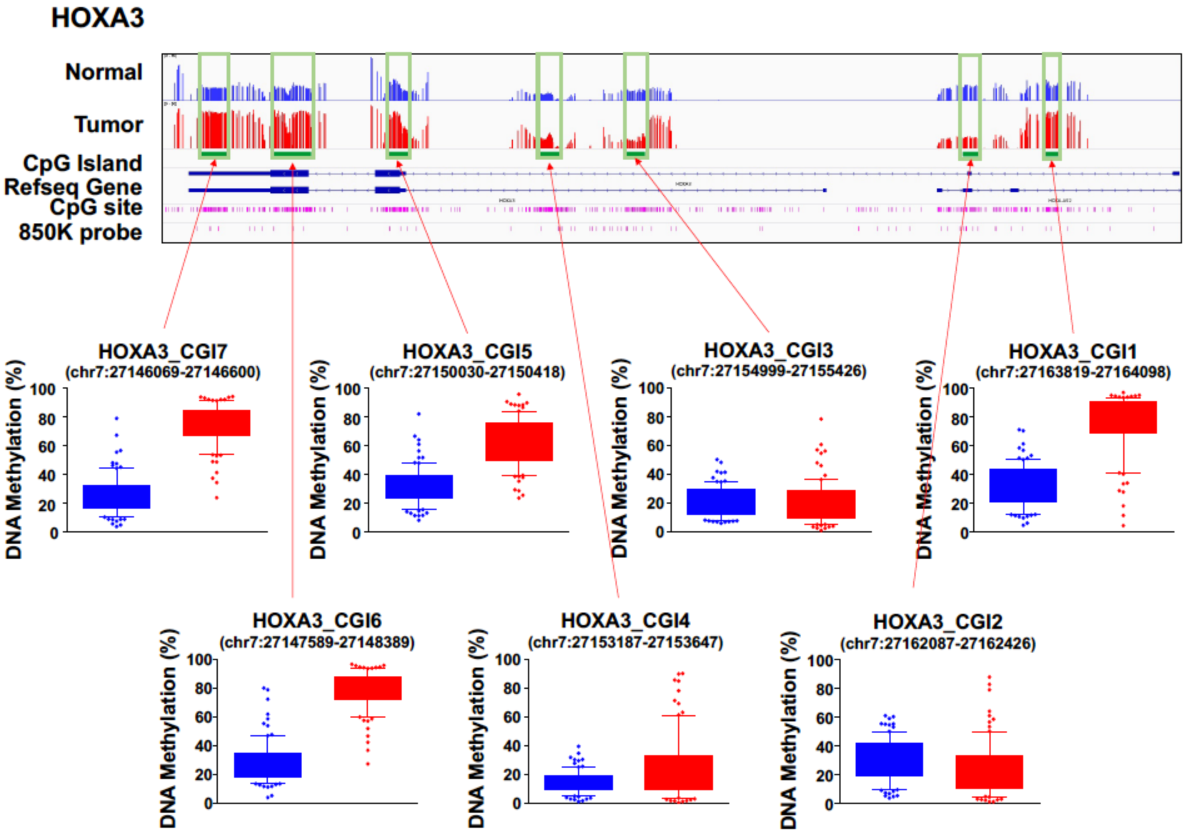

Supplementary Fig. 10 Representation of methylation patterns in HOXA3-related CpG islands from generated targeted bisulfite sequencing data.

The DNA methylation level of 7 CpG islands in HOXA3 (chr7:27,163,819-27,164,098, chr7:27,162,087-27,162,426, chr7:27,154,999-27,155,426, chr7:27,153,187-27,153,647, chr7:27,150,030-27,150,418, chr7:27,147,589-27,148,389, chr7:27,146,069-27,146,600) according to generated targeted bisulfite sequencing data. The human genome reference version used was hg19, and data were visualized using the IGV browser. The bar graph indicates the average methylation level of CpG sites in each CpG island.

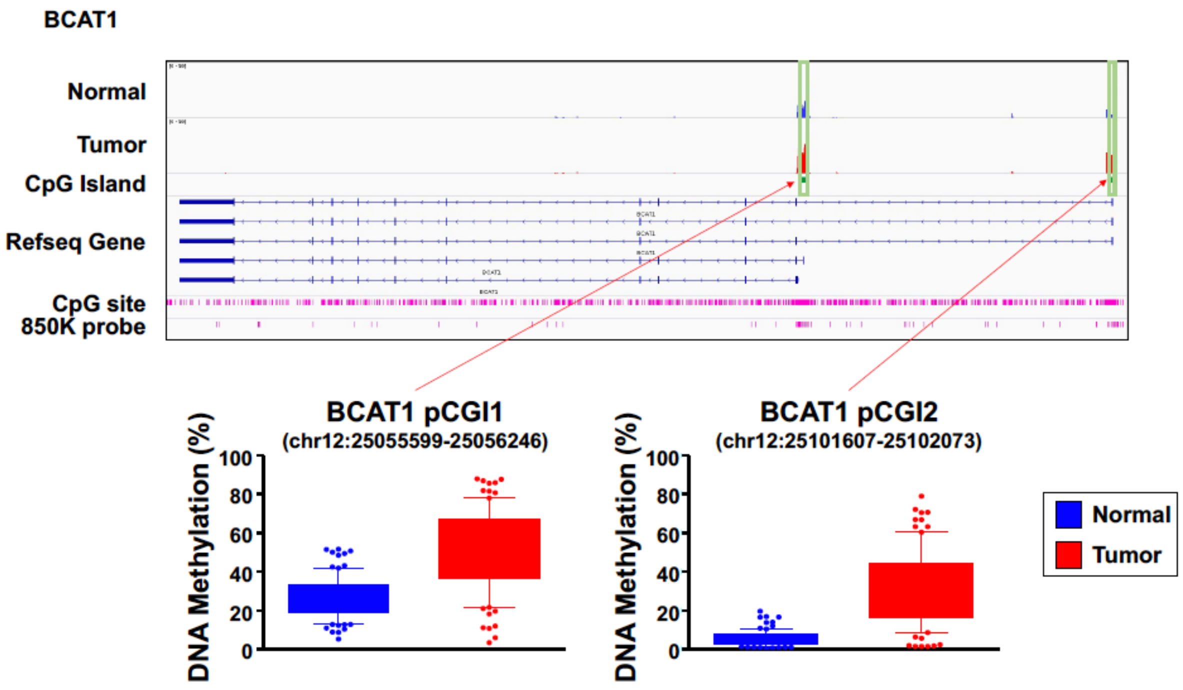

Supplementary Fig. 11 Representation of methylation patterns in BCAT1-related CpG islands from generated targeted bisulfite sequencing data.

DNA methylation levels of two CpG islands in BCAT1 (chr12:25,101,607-25,102,073, chr12:25,055,599-25,056,246) according to generated targeted bisulfite sequencing data. The human genome reference version used was hg19, and data were visualized using the IGV browser. The bar graph indicates the average methylation level of CpG sites in each CpG island.

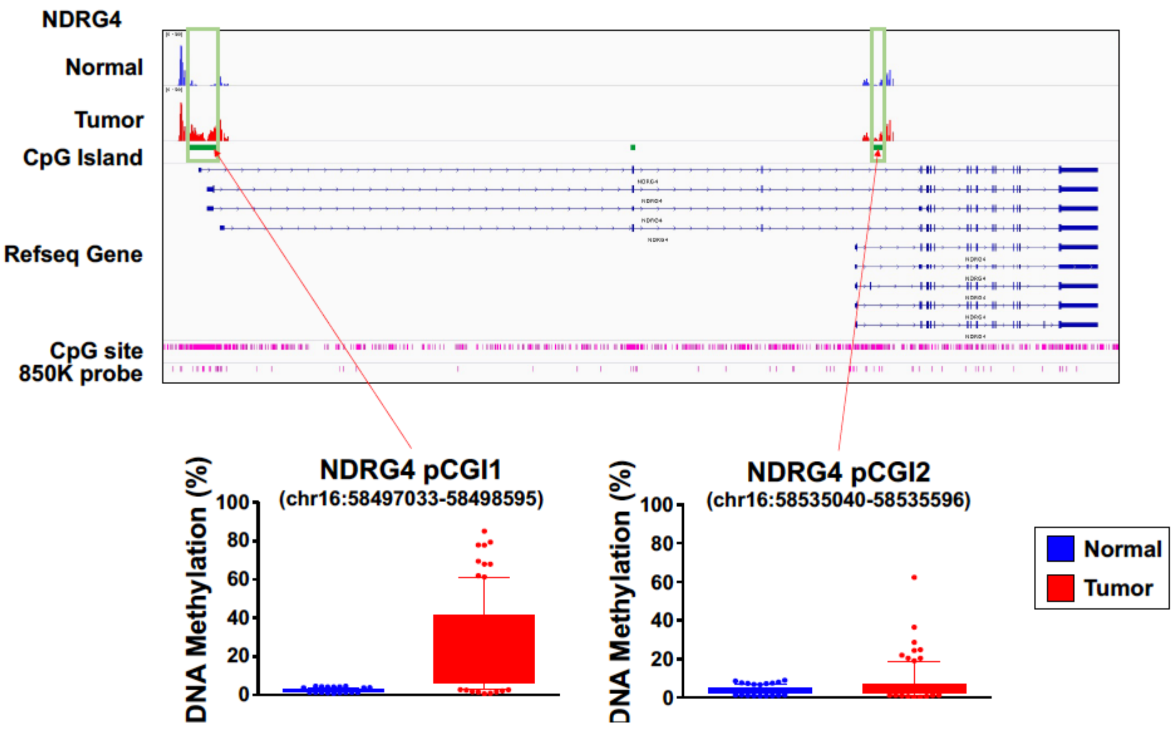

Supplementary Fig. 12 Representation of methylation patterns in NDRG4-related CpG islands from generated targeted bisulfite sequencing data.

DNA methylation levels of two CpG islands in NDRG4 (chr16:58,497,033-58,498,595, chr16:58,535,040-58,535,596) according to generated targeted bisulfite sequencing data. The human genome reference version used was hg19, and data were visualized using the IGV browser. The bar graph indicates the average methylation level of CpG sites in each CpG island.

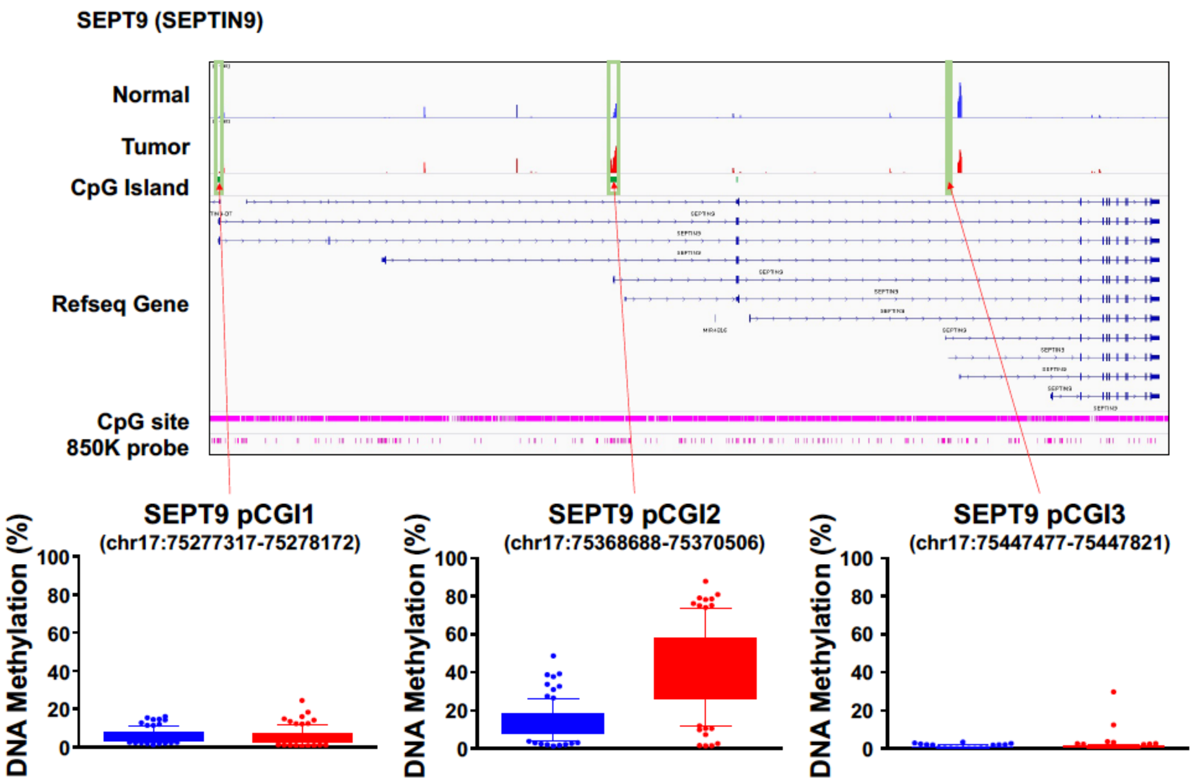

Supplementary Fig. 13 Representation of methylation patterns in SEPT9-related CpG islands from generated targeted bisulfite sequencing data.

DNA methylation levels of three CpG islands in SEPT9 (chr17:75,277,317-75,278,172, chr17:75,368,688-75,370,506, chr17:75,447,477-75,447,821) according to generated targeted bisulfite sequencing data. The human genome reference version used was hg19, and data were visualized using the IGV browser. The bar graph indicates the average methylation level of CpG sites in each CpG island.

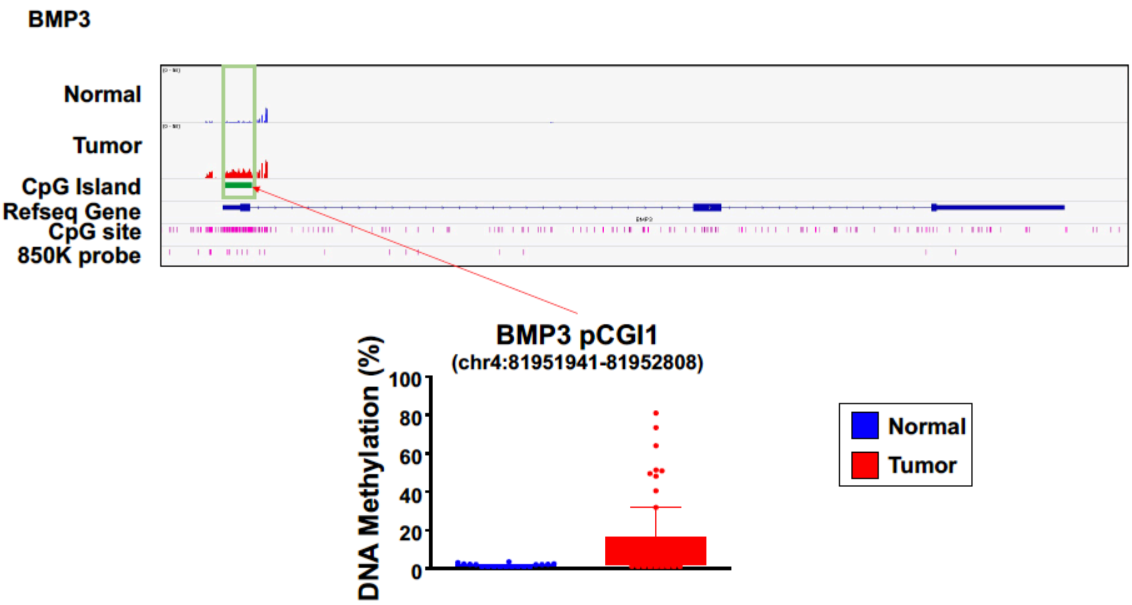

Supplementary Fig. 14 Representation of methylation patterns in BMP3-related CpG islands from generated targeted bisulfite sequencing data.

The DNA methylation level of a promoter CpG island in BMP3 (chr4:81,951,941-81,952,808) according to generated targeted bisulfite sequencing data. The human genome reference version used was hg19, and data were visualized using the IGV browser. The bar graph indicates the average methylation level of CpG sites in each CpG island.

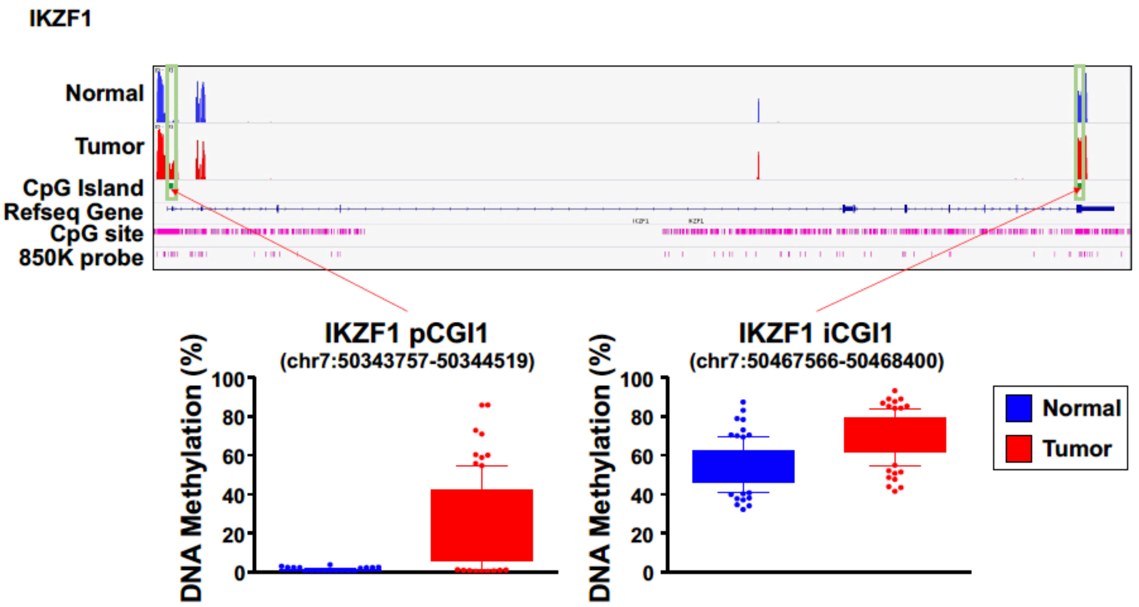

Supplementary Fig. 15 Representation of methylation patterns in IKZF1-related CpG islands from generated targeted bisulfite sequencing data.

The DNA methylation levels of two CpG islands in IKZF1 (chr7:50,343,757-50,344,519, chr7:50,467,566-50,468,400) according to generated targeted bisulfite sequencing data. The human genome reference version used was hg19, and data were visualized using the IGV browser. The bar graph indicates the average methylation level of CpG sites in each CpG island.
